# Toward an Integrated Model of Supportive Peer Relationships in Early Adolescence: A Systematic Review and Exploratory Meta-Analysis

**DOI:** 10.3389/fpsyg.2021.589403

**Published:** 2021-02-25

**Authors:** Marija Mitic, Kate A. Woodcock, Michaela Amering, Ina Krammer, Katharina A. M. Stiehl, Sonja Zehetmayer, Beate Schrank

**Affiliations:** ^1^D.O.T. Research Group for Mental Health of Children and Adolescents, Ludwig Boltzmann Society at Karl Landsteiner University of Health Sciences, Krems on the Danube, Austria; ^2^Centre for Applied Psychology, School of Psychology, University of Birmingham, Birmingham, United Kingdom; ^3^Clinical Division of Social Psychiatry, Department of Psychiatry and Psychotherapy, Medical University of Vienna, Vienna, Austria; ^4^Centre for Medical Statistics, Informatics and Intelligent Systems, Medical University of Vienna, Vienna, Austria; ^5^Department of Psychiatry and Psychotherapy, University Hospital Tulln, Karl Landsteiner University of Health Sciences, Tulln, Austria

**Keywords:** early adolescence, friendship, integrated model, loneliness, meta-analysis, peer relationships, social-emotional well-being, peer relationship quality

## Abstract

Supportive peer relationships (SPR) are crucial for mental and physical health. Early adolescence is an especially important period in which peer influence and school environment strongly shape psychological development and maturation of core social-emotional regulatory functions. Yet, there is no integrated evidence based model of SPR in this age group to inform future research and practice. The current meta-analysis synthetizes evidence from 364 studies into an integrated model of potential determinants of SPR in early adolescence. The model encompasses links with 93 variables referring to individual (identity, skills/strengths, affect/well-being, and behavior/health) and environmental (peer group, school, family, community, and internet/technology) potential influences on SPR based on cross-sectional correlational data. Findings suggest the central importance of identity and social–emotional skills in SPR. School environment stands out as a compelling setting for future prevention programs. Finally, we underscore an alarming gap of research on the influence of the virtual and online environment on youth's social realm given its unquestionable importance as a globally expanding social interaction setting. Hence, we propose an integrated model that can serve as organizational framework, which may ultimately lead to the adoption of a more structured and integrated approach to understanding peer relationship processes in youth and contribute to overcoming marked fragmentation in the field.

## Introduction

### A Role of Supportive Peer Relationships in Early Adolescence

Adolescents' social realm encompasses relationships with family members, peers, school staff, and people in any other physical (e.g., neighborhood; Karcher and Sass, 2010) or virtual community (Nesi et al., 2018a,b) to which an individual belongs. Some of these relationships reflect the norms of the adult world (conventional types—e.g., relationships with parents and teachers). Importantly however, peer relationships (PR) are linked to unsupervised activities and norms of the adolescent world (Karcher et al., 2008) and thus possess a specific character that must be understood independently from the other social contexts.

From a developmental perspective, the family plays a central role in the life of a child. However, in middle childhood and early adolescence the influence of PR on social and emotional outcomes intensifies (Somerville, 2013) and these relationships become one of the key factors in shaping and directing young people's psychological development (Barnes et al., 2007). Evidence from cognitive neuroscience suggests that this period may be critical for the maturation of complex socio-emotional and cognitive skills that influence later mental health outcomes (i.e., development of the so-called “social brain”—Lamblin et al., 2017; Wong et al., 2018). Indeed, in line with an important role of PR in these aspects of social emotional development, supportive peer relationships (SPR, i.e., relationships, of high perceived quality, which serve a protective function for an individual), contribute to the sense of self (Harter, 1999; Meeus et al., 2002), social competence (Laible, 2007), and academic performance (Vandell and Hembree, 1994; Schwartz et al., 2008). Furthermore, a lack of SPR is linked to a range of negative outcomes, including social withdrawal (Bond et al., 2007), risk-taking behavior such as early commencement of smoking, drinking, and sexual relationships (Kipping et al., 2012), increased risk of juvenile delinquency (Wasserman et al., 2003) and long-term metal health consequences (Lereya et al., 2015).

In light of the fact that SPR pervade healthy functioning, an integrative understanding of early adolescents' SPR is necessary to enable effective and sustainable preventive and therapeutic strategies relevant for the aforementioned outcomes. This requires SPR to be defined in a manner meaningful and informative for future research and practice as there is somewhat conflicting evidence on the relative importance of different aspects of PR—quality, popularity, and stability (Gifford-Smith and Brownell, 2003; Flannery and Smith, 2017) that contributes to the existing fragmentation in the field of PR (Brown and Larson, 2009). We believe that these conflicts can be reconciled by recognizing that whilst early social relationships tend to be instable—which links to the concept of popularity (LaFontana and Cillessen, 2010)—even one peer relationship of high perceived quality (i.e., a mutual friendship) can be protective against the feeling of loneliness (Sanderson and Siegal, 1995), thus it is the perceived quality of the peer relationship by the individual who is potentially supported by that relationship, which is the aspect of interest. In contrast to peer relationship quality, early changes in status and/or in the number of friendships can be viewed as indicators of the development of a more complex understanding of the social realm, individual bonding needs and preferences, and as such do not necessarily relate to *supportive* peer relationships (Gifford-Smith and Brownell, 2003; Flannery and Smith, 2017). As such, we offer peer relationship quality (PRQ) as the most appropriate integrative and quantifiable concept for studying SPR, with PRQ being an individual's perception of the quality of a peer relationship (see section Materials and Methods for the operational definition). Further, we will refer to our work as integrative (Merriam Webster—having different parts working together as a unit) as we aspire to overcome the existing fragmentation (Merriam Webster—separation into distinct parts) in the field of PR.

In summary, SPR in early adolescence are one of the crucial factors for health and well-being both in the short- and in the long-term. However—as we will demonstrate below—there is currently no integrative evidence based model of SPR in this age group to inform intervention strategies (Brown and Larson, 2009). The current review aims to address this need and to initiate the development of such model by drawing on evidence of the diverse influences on PR from the scientific literature.

### Theoretical Models of Human Development in Interpersonal Contexts

Numerous established theories have studied the process of human development in diverse interpersonal contexts. Attachment theory examined the complex paths of human development in relationships with caregivers (Bowlby, 1969); whereas, interpersonal theory studied how relationships with “significant others”—especially peers (Sullivan, 2013) complement each other in developmental processes. Indeed, the cumulative interpersonal risk model (Epkins and Heckler, 2011) stipulates the complementary roles these relationships have in the genesis of affective disorders. Yet, this complementarity also enables a mutual buffering effect (resilience theory; Zimmerman, 2013); thus, PR can buffer against adversity in the family and vice versa. However, all relationships are inherently embedded into specific environmental contexts and they need to be understood in relation to these contexts.

Bronfenbrenner's bioecological model (Bronfenbrenner and Morris, 2007) puts relationships in the context of ecological systems, stipulates the environmental context as a powerful determinant of human development and considers the reciprocal interactions between different systems connected via social interactions. Yet, the model fails to account for precise relationships between the systems and focuses more on spatial than on interpersonal dimensions of ecological systems (Neal and Neal, 2013).

Magnusson's holistic-interactionistic model (Magnusson, 2001) draws on the bioecological model and focuses on the person in the context and introduces an idea of an individual as an organism and its physical environment as a system that functions as an organized whole. Psychological events reflect two interdependent levels of continuously ongoing individual processes: (a) those that interact between individual and environmental factors, and (b) those among psychobiological and behavioral components in the individual (Magnusson and Håkan, 2007). Hence, the integrated holistic nature of developmental processes implies that they cannot be decomposed into or understood as independent components. The model challenges traditional, variable-oriented unidirectional psychological research models and proposes a functional interaction model based on principles of reciprocity, interdependence, and non-linearity of interactions between and within the individual and its environment.

By applying the functional interaction principles to PR, it becomes obvious that the current developmental context and aspect of the self that emerge from it determine the quality of the current and future relationships in a given environmental context and short- and long-term mental health outcomes. Whereas, the developmental mechanisms in the context of relationships with caregivers are frequently studied and relatively integrated (e.g., Stormshak, 2009), the integrative understanding of the PR is missing. Furthermore, research on PR is fragmented and grounded in traditional socialization theories based on unidirectional models that fail to capture the reciprocal, translational nature of influences *in* and *on* PR (Brown and Larson, 2009) and their the role in promoting well-being remains mainly unknown. The complex interplay of individual, interpersonal, and sociopolitical factors models a dynamic system that imposes great challenges for empirical research on PR (Magnusson and Håkan, 2007).

We aim to develop of a flexible framework based on the principles of holistic functional interactionism that allows integrated understanding of SPR and surrounding environmental systems. The integrated model is a critical step toward overcoming existing fragmentation in the field of PR.

### Integrative Approach Toward Advancing Interventions That Foster Supportive Peer Relationships and Well-Being

The above described fragmentation is mirrored in the interventions in the field. Although experts agree that complex ecological strategies are necessary to deliver the expected impact in the field of youth mental health (Tolan, 2016; Ross and Tolan, 2018), complex interventions usually comprise multiple components, which may act independently or interdependently, with the “active ingredient(s)” often being difficult to specify (Pfadenhauer et al., 2017). Such interventions challenge current approaches to conceptualization, assessment, and implementation.

The realist approach suggests a framework adaptation toward top-down understanding of active ingredients, their interactions with the delivery context and the resultant development of ecologically valid models (Fletcher et al., 2016) during the evaluation of complex interventions. However, many of the widespread intervention strategies have not been empirically validated or rooted in comprehensive evidence based theoretical models (e.g., Arnold and Silliman, 2017; Ciocanel et al., 2017; Arnold and Gagnon, 2019). Indeed, during the development of complex interventions a comprehensive (bottom-up) understanding of the role SPR have in promoting well-being, in addition to mapping potential determinants and their mechanisms from empirical studies, is another missing link crucial for overcoming fragmentation in and between theoretical and interventional research.

In summary, given the interdependence between the SPR and well-being, integrated bottom-up understanding of SPR may help to advance interventions in the field of youth mental health. The current model is a first step toward such integration, aiming to inform the design, implementation, and interpretation of empirical studies on SPR in early adolescence. It aims to spark further (qualitative and qualitative) research toward integrated understanding of individual, interpersonal, and sociopolitical developmental contexts, and to inform the theory driven development of new and refinement of existing interventions. Notably, since our aim is to inspire future research that will ultimately inform on an integrated understanding of the determinants of SPR and pathways to intervention, we take the deliberate stance of referring to potential determinants of SPR throughout this work. Whilst the data we draw on do not allow us to assert a causal direction (they are correlational data), this approach allows us to present a coherent theoretical model, which can be examined directly in future empirical research.

## Materials and Methods

### Systematic Search

In order to study SPR, we focus on PRQ, which we define as any aspect (positive or negative) of the quality of relationships as perceived by an individual, with at least one specific peer or with peers in general—including for example, intimacy, value, supportive function, closeness, or (negative aspects) lack of any of these. Potential determinants of SPR in early adolescents are considered as factors that may influence PRQ. Hence, any variable (e.g., psychological or environmental) correlated with PRQ in this population was considered for inclusion. Initially, we placed no restrictions on measurement instruments.

The systematic review was conducted according to a structured protocol registered on the PROSPERO repository (CRD42018107945). The systematic search included five databases (ERIC, EMBASE, MEDLINE, PsycINFO, and Cochrane Library) and gray literature sources (OSF Preprints and OpenGrey), searched from inception until end of December 2017 using widely defined terms for the early adolescent population (“child^*^”, “adoles^*^”, “teenage^*^”, “youth^*^”, “young^*^”) combined with a set of terms for the PRQ (“peer support”, “emotional support”, “emotional connection”, “social network(s)”, “social relation(s)”, “social relationship(s)”, “social connection(s)”, “social connectivity”, “social connectedness”, “belongingness”, “loneliness”, “social isolation”, “social acceptance”, “social withdrawal”, “friendship(s)”, “friend(s)”, “peer relation(s)”, “peer relationship(s)”, “peer connection(s)”, “peer connectedness”, “well-being”). Studies in English, German, Portuguese, Spanish, Italian, Serbian, Croatian, and Hebrew were considered for inclusion. An additional hand search included the first 200 references identified via Google Scholar, as well as reference lists and cited reference searches of included studies and relevant literature reviews (Laursen and Hartl, 2013; Newsom et al., 2013; Pallini et al., 2014; Li and Wong, 2015; Meter and Card, 2016). The detailed search strategy, including search terms is available in the online supplement.

### Eligibility Criteria

#### Inclusion Criteria

Cross-sectional and longitudinal correlational studies that measured some aspect of PRQ in early adolescents (aged 8–14 years) were included. Studies must include a full- or subscale on PRQ as defined by the authors of the measure (i.e., PRQ factor measured as a part of social support scale), with available reliability and/or validity information and its correlation with at least one psychological, social, or environmental factor. Due to the large number of eligible studies and questionable translatability of dated publications to the current societal context, we only included articles published after 1999.

#### Exclusion Criteria

We excluded studies with the following populations: delinquent and homeless; emigrational backgrounds including refugees; sexual, religious, and ethnic minorities; intellectual, physical, or sensory disabilities; acute or chronic physical illness; psychiatric clinical samples with diagnosed affective disorders, bipolar disorder, psychotic disorders, eating disorders, attention deficit hyperactivity disorder, or conduct disorder. Characteristics of such special populations create specific developmental contexts for SPR during early adolescence (e.g., Dunn, 2004; Bos et al., 2008; Korkiamaki, 2011; Shiffman et al., 2016). The influence of these contexts on SPR is critical to understand, particularly when considering how to support the most vulnerable members of society. However, to understand such influence in a systematic way that will be productive for future research; we need a framework that comprehensively describes potential determinants of SPR in early adolescents on which to build. We therefore excluded such populations from the present review.

During the quantitative data analysis, additional exclusion criteria were established to enable meaningful statistical analysis of the identified studies and to obtain results pertinent to early adolescence. Initially, we placed no restrictions on the mean age of participants as long as the age range showed an overlap with our target range (8–14 years). However, due to the wide age range in some studies, which may have limited the relevance to the early adolescent period, we excluded studies in which the mean age exceeded 15 years, this upper age limit was established based on the highest age limit used to describe early adolescence identified in the literature (Sawyer et al., 2018). Six studies with a mean age of 16 were included to enable meaningful quantitative analysis for certain variables. However, the mean age of the analyzed sample was 12.5 years. Furthermore, on the variable level only 18.3% of variables had samples with the mean age over 14 years. Information on the applicability to the context of early adolescence for each variable is available in the results tables.

Our aim was to bring together all potential determinants of early adolescent SPR in a single model, to act as a basis for further research. A minority of studies eligible for meta-analyses 10.9% included longitudinal data. Longitudinal data can provide stronger evidence that a potential determinant is a true determinant. However, such data set is limited by the greater pragmatic demands on longitudinal research. For the present goal to develop an integrated model, longitudinal correlations were therefore excluded. An additional challenge was the marked heterogeneity in the measurement instruments for potential determinants. Our aim to develop an integrated framework and our focus on adolescents' experiences demanded a coherent quantitative analysis and comparable measurements, hence for variables that were assessed with diverse procedures (e.g., victimization assessed via self-report and peer nominations) we prioritized psychometric self-reported measures for inclusion in the meta-analysis, and this led to the exclusion of studies that used only sociometric and/or observational instruments. Finally, studies that reported only correlations controlled for confounders or aggregated correlations for multiple assessment points were excluded if authors were unreachable or non-responsive to our requests for additional information. Again, such data would not allow meaningful quantitative analysis if analyzed together with the cross-sectional uncontrolled time points. Information on exclusion from the meta-analysis with reasons can be found in the Supplementary Table 1.

### Study Selection and Data Extraction

The identification, screening, and data extraction of eligible studies was conducted in accordance with the PRISMA statement (Moher et al., 2010). Two reviewers independently screened the identified references. Disagreements over the eligibility of particular studies were resolved through discussion with a third reviewer. A standardized form was used to extract data for the assessment of study quality and evidence synthesis. Due to the large number of eligible studies, data were extracted by multiple trained reviewers and controlled by an experienced researcher. To establish consistency between multiple reviewers, supervised training was provided. During the training period reviewers extracted data independently, compared extractions, and resolved discrepancies through discussion. This process was continued until concordance was present in 10 consecutive independent extractions.

### Quality Appraisal

The methodological quality of included studies was assessed in accordance with recommendations from the recent systematic review by Zeng et al. (2015), which informed our choice of the assessment instruments. The Newcastle-Ottawa Scale (NOS; Stang, 2010) was used to rate quality of longitudinal studies. Cross-sectional studies were rated using the checklist recommended by Agency for Healthcare Research and Quality (AHRQ; Zeng et al., 2015). Given both instruments were designed to assess methodological quality of clinical studies; scales were adapted for the purpose of our research for the panel studies design. The adapted instruments comprised 10 (ARHQ) and 7 (NOS) distinct criteria. To enable meaningful interpretation of the results numeric scores (higher score indicates higher risk of bias) were defined and studies were judged as having low (≤1), moderate (≤2), or high (≥2,5) risk of bias. The adapted instruments and assessment criteria are available in the study supplement (Supplementary Document 2).

The methodological quality of included studies was assessed independently, by two reviewers in 213 (59%) studies, the rest of the included studies were assessed by a single trained reviewer. Disagreement between the assessors was resolved in discussion with involvement of a third experienced researcher where necessary. The level of reliability between the reviewers was fair (McHugh, 2012)—Cohen's kappa (k) 0.44.

### Qualitative Analysis

The extracted data were imported to QSR NVivo (Richards, 2005) for the initial qualitative analysis. For every study, a unique case was created along with assigned attributes including authors' names, study design, year of publication, country, methodological quality, and mean age of study participants. Next, two independent reviewers coded potential determinants measurements and measures of PRQ in parallel. Reviewers consulted and discussed the coding procedures on a daily basis including an experienced supervisor when needed.

Measures of PRQ were examined on the dimensional level to guide the development of the analysis strategy. However, the heterogeneity among instruments was marked. Furthermore, reporting was not consistent: whereas some authors provided results on a subscale level, others reported aggregated scores for multiple subscales. Hence, to be able to perform meaningful statistical analysis, we decided not to differentiate between the different measures of PRQ and their sub-dimensions or between positive and negative dimensions. This decision was based on the assumption supported by the prevailing literature that positive and negative quality aspects of PR are the opposite polarities of the same continuum (Brown and Larson, 2009). Therefore, we analyzed positive dimensions (such as peer attachment and positive friendship qualities) and negative dimensions (such as loneliness and negative friendship qualities) together. To enable the quantitative analysis, correlations relating to negative dimensions of PRQ were reversed so that constructs could be considered in positive terms. For example, reported positive correlates of loneliness were considered as negative correlates of absence of loneliness, which is comprised in our definition of PRQ. The same method was applied in cases where correlates were judged to be the opposite polarities of the same construct (e.g., hope and hopelessness). All decisions were made based on the consensus between leading and supervising author.

**Figure 1:**
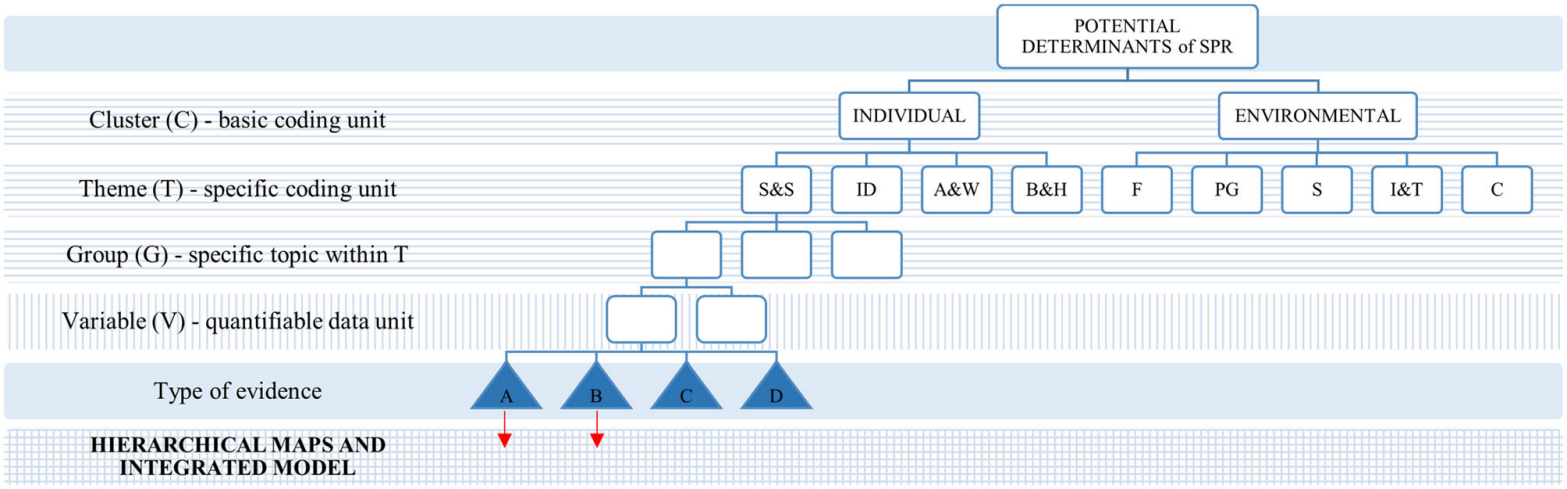
Coding hierarchy and model development process. AandW, Affect and well-being; BandH, behavior and health; C, community; F, family; IandT, internet and technology; PG, peer group; S, school; SandS, skills and strengths. 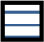 Qualitative analysis; 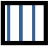 Meta-analysis; 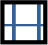 Mixed methods; 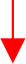 Types of evidence used for the model development (see Table 1).

Based on the identified potential determinants constructs, a coding hierarchy (Figure 1) was inductively developed. The data were then hierarchically clustered into first-order concepts that include clusters (C), themes (T), groups (G), and variables (V), as depicted in Figure 1. Finally, for each of the variables, the quantitative data were extracted into a pre-structured table to enable the statistical analysis. The extraction process was performed by trained reviewers and controlled throughout by an experienced reviewer. Missing data from included studies were requested from corresponding authors with limited success (10%).

**Table 1:** Types of evidence.

Strong	Type A+	*ß ≥ |+/−0.40|*
Moderate	Type A	*ß ≥ |+/−0.30|*
Small	Type B	*ß ≥ |+/−0.20|*
Very small	Type C	*ß ≥ |+/−0.10|*
Negligible	Type D	*ß ≤ |+/−0.10|*

*ß, estimated correlation coefficient; |+/– x|, the absolute value*.

*All scores are statistically significant at the 95% confidence level. Statistically non-significant scores were classified as negligible*.

### Meta-Analysis

Meta-analyses based on bivariate correlation coefficients were performed by a statistician. For the effect size estimation the Fisher z transformation (Fisher, 1921) was applied to obtain approximately normal distributions of the test statistics, for the presentation of the results the effect sizes were reconverted to correlation coefficients. For the analyses, repeated correlation coefficients within the publications have to be considered because the assumption of independence may be violated. Two different approaches were applied—conservative and mixed effects approach (Viechtbauer, 2010). First, for all publications, the Fisher transformed correlation coefficients as well as the sample sizes were averaged. Second, a mixed effects model was calculated, where random effects were introduced. For both approaches the estimated correlation coefficient (ß) of the meta-analysis, the corresponding 95% confidence intervals and a *p*-value, which tests the null hypothesis (ß = *0*), were computed. Both approaches yielded equivalent results and thus we report the results of the mixed effects model. Forest plots showing the correlation coefficients of the single studies together with the 95% confidence intervals and the corresponding resulting meta-analysis estimators were generated and are available in the study supplement along with a histogram of the conducted meta-analysis correlation coefficients (Supplementary Figures 2–95). Finally, the statistical heterogeneity index (*I*^2^) that quantifies the degree of inconsistency across the studies' results was calculated (Viechtbauer, 2010). For the analyses, the R programming language (Team, 2019) and the “metafor package” (Viechtbauer, 2010) were applied, with aim to answer the following research question: What are potential determinants of SPR in early adolescence?

To facilitate the mapping of salient findings we defined 5 types of evidence (Table 1) following recommendations from (Cohen, 2013). The evidence types were defined based on estimated correlation coefficient (ß) and *p*-value. The variables showing small to strong estimated correlation coefficients (i.e., evidence type A and B) were included in the model development, which employed a mixed-methods synthesis.

### Mixed Methods Approach to Model Development

The mixed methods synthesis was informed by techniques described in Popay et al. s' guidelines (2006) that were adapted for the purpose of the current study. The methodology was based on the concept mapping method (Mulrow et al., 1997) which involves linking multiple pieces of evidence from across individual studies (variables in the current context) to a model that highlights key concepts or issues relevant to the research question and represents relationships between these. This approach uses diagrams and flow charts to visually represent studied concepts and relationships between them and we used it to develop the hierarchical maps of potential determinants. Further, the conceptual triangulation method (Foster, 1997) was adapted (Supplementary Figure 1) to inform the model development by combining our qualitative and quantitative findings with recognized theoretical models that serve as a base for the definition of the second-order concepts. This allowed us to generate the integrated model through which the SPR may be better understood on the basis of the diverse sources of synthesized evidence.

First, the quantitative data (tabulated variables) were examined with respect to strength and direction of association with PRQ. The variables showing salient findings were embedded in the existing qualitatively derived coding hierarchy of first-order concepts and named to reflect positive association with PRQ (i.e., high emotion regulation ability and low depression show moderate positive association with PRQ) to develop the hierarchical maps of evidence and explore relationships between the concepts. Second, the hierarchical maps were examined in parallel with the recognized theoretical models to develop the integrative second-order concepts that embed the salient findings in the recognized theoretical models with respect to identified relationships between the concepts. Hence, the first-order concepts individual factors were transformed in the second-order concepts—self-building-blocks based on Magnusson's holistic-interactionistic model (Magnusson, 2003; Magnusson and Håkan, 2007) and the environmental factors (first-order) were transformed in the environmental planes (second-order) based on Bronfenbrenner's ecological model (Bronfenbrenner and Ceci, 1994; Bronfenbrenner and Morris, 2007). Finally, the evidence based integrated model of SPR was developed and graphically presented.

### Publication Bias

There is evidence on the pervasive publication bias in the entire field of psychology (Kühberger et al., 2014) that may arise due to non-publication of negative findings, selective reporting, or misinterpretation of results (Devito and Goldacre, 2019). Hence, in order to minimize the publication bias we followed the recommendations from Devito and Goldacre (2019) and (a) conducted an extensive literature search including gray literature sources, (b) included primary, secondary and exploratory endpoints in our analyses, (c) requested missing data from authors, and (d) performed three different sensitivity analyses. First, two funnel plots (scatter plot of the standard error and the effect size i.e., the Fisher transformed correlation coefficients) for each meta-analysis were generated, one for the mixed model approach and one for the conservative mean approach. In these analyses, an asymmetrical funnel plot would indicate the presence of publication bias. Second, for the conservative approach we applied the trim and fill method—a nonparametric, rank-based data augmentation technique proposed by Duval and Tweedie (2000a,b) that estimates the number of studies missing from a meta-analysis due to the suppression of the most extreme results on one side of the funnel plot and recomputes effect sizes and *p*-values. Third, for the conservative and the mixed data a rank correlation test for funnel plot asymmetry as described by Begg and Mazumdar (1994) was performed. The test is used to examine the association between the observed outcomes and the corresponding sampling variances. A high correlation indicates that the funnel plot is asymmetric, which may be a result of publication bias.

## Results

### Characteristics of Included Studies

**Figure 2:**
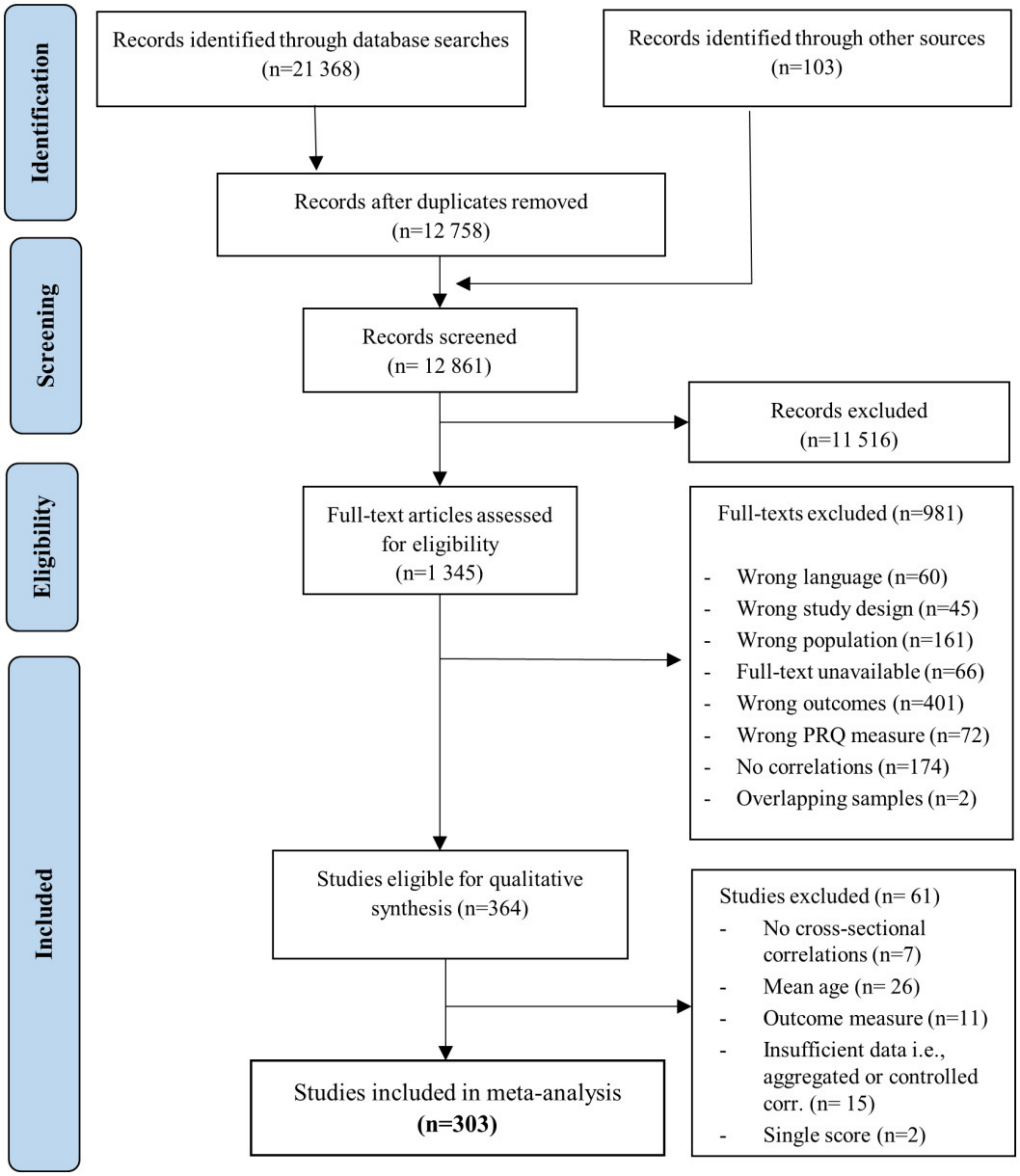
PRISMA flow diagram. Diagram of the study inclusion process adapted from Moher et al. (2010).

We identified 364 studies from 361 publications eligible for inclusion in the narrative synthesis: 260 studies from systematic database search, 99 studies from reference search and 4 studies from hand-search in Google scholar (Figure 2). Included articles were published between 2000 and 2018. With respect to geographical region, 50.1% (183) of eligible studies had samples from North America (including USA and Canada), 25.2% (92) from Western Europe, 17.3% (63) from Asian countries, 5.8% (21) from Australia and New Zealand, and 1.6% (6) from Central or South America. The majority (97%) of the included studies had samples comprised of typically developing school-aged populations. A few studies included participants from vulnerable populations such as children of parents with a mental illness (Fraser and Pakenham, 2009; Festa and Ginsburg, 2011), foster care populations (Merritt and Snyder, 2015; Thompson et al., 2016), or socio-economically disadvantaged populations (Salzinger et al., 2011; Tanigawa et al., 2011; Goodearl et al., 2014; Lamis et al., 2014; Oldfield et al., 2016; Wentzel et al., 2016; Guay et al., 2017). With respect to study design, 36.4% (133) had a longitudinal and 63.6% (232) a cross-sectional design. We included 303 studies in the meta-analyses. The mean age of the analyzed sample was 12.5 (age range 8.09–16.41; SD = 1.8). The Supplementary Table 1 contains information on study characteristics including: first author information, year of publication, design, country in which the study was conducted, age of participants, risk of bias, PRQ measure type, data on in/exclusion from the quantitative analysis with reasons. The reference list of included studies is also available in the online supplement – Supplementary Document 3.

#### Methodological Quality of Included Studies

The methodological quality was generally low: 17 studies (4.7%) had a moderate risk of bias and 348 (95.3%) had a high risk of bias. Out of 17 studies with moderate risk of bias, 10 had longitudinal, and 7 had cross-sectional designs. Given this is an exploratory analysis that aims to obtain a comprehensive picture of potential determinants of SPR, we decided not to exclude papers based on their methodological quality. However, we consider the general low methodological quality in our interpretation of the derived evidence. The detailed information on methodological quality for individual studies is available in the Supplementary Table 1.

#### Publication Bias

Except dissertations, we failed to identify additional unpublished studies. The missing data were requested from corresponding authors for 141 and received for 16 studies. Visual and quantitative evaluation of the sensitivity analyses' results showed consistent small to irrelevant publication bias in the 303 studies included in the meta-analyses. During the visual evaluation of funnel plots (Supplementary Figures 96–219), no significant asymmetry was observed. Trim and fill and rank correlation tests yielded small/irrelevant adjustments in values across all variables after controlling for publication bias. Hence, there is no evidence of publication bias being a major issue for the present metal-analyses.

### Measures of Peer Relationship Quality

We identified 87 distinct self-reported measures of PRQ. Detailed information on measurement instruments from individual studies is available in the Supplementary Table 1. The identified quality dimensions are intimacy, closeness, relatedness, support, attachment, belonging and loneliness—and these are measured in relation to best friend, close friends (same or opposite sex), peers or classmates. With respect to the structure of used measures, some studies employed instruments with mixed sub-dimensions referring to positive and negative qualities e.g., the Friendship Quality Questionnaire (FQQ; Parker and Asher, 1993), Network of Relationships Inventory (NRI; Furman and Buhrmester, 1985), Inventory of Parent and Peer Attachment (IPPA; Armsden and Greenberg, 1987), and the Loneliness and Social Dissatisfaction Questionnaire (LSDQ; Asher et al., 1984). Other authors employed measures exclusively referring to positive qualities such as the Child and Adolescent Social Support Scale (CASSS; Malecki and Demaray, 2002). Finally, measures with negative qualities such as the Peer Network and Dyadic Loneliness scale (PNDL; Hoza et al., 2000) and the University of California Los Angeles Loneliness Scale (UCLA; Russell, 1996) were used in some studies. Many authors failed to provide detailed information regarding employed instruments and/or their modifications; hence, in many cases it was not possible to draw conclusions about the exact structure of employed instruments.

### Potential Determinants of Peer Relationship Quality

We identified two clusters of potential determinants of PRQ in early adolescence—individual and environmental (see Figure 1). Within each of the two clusters, we defined specific themes referring to (a) individual skills and strengths, (b) identity aspects, (c) behavior and health, (d) affect and wellbeing, (f) family, (g) peer group, (h) school, (i) internet and technology, and (j) community. Within the themes, we defined specific topics—groups. Each group comprised multiple quantifiable data units—variables. In total, we computed 93 variables (55 in the individual and 38 in the environmental cluster), resulting in total of 2,097 correlation coefficients used for statistical analysis. The minimum number of correlation coefficients per variable was 2, the maximum 193 (mean 22). Some publications appear several times as different scores were used for the analysis; however, none of these scores were included more than once. The minimum number of studies per variable was 2, the maximum number was 73. The variables showing A or B type of evidence (i.e., ß ≥ 0.20; 45.5% of all analyzed variables) informed the development of our integrated model of possible determinants of SPR in early adolescence. The model and its implications for research and practice will be described in more detail in the discussion section; here we focus on individual variables to obtain a comprehensive overview of identified factors.

#### Individual Cluster

##### Skills and Strengths

This theme contains 7 distinct groups: (a) emotion regulation, (b) social cognition, (c) social skills, (d) cognitive and language skills, (e) academic skills, (f) emotional intelligence, and (g) autonomy in decision making; and 12 different variables referring to individual skills and strengths (Table 2). Amongst skills variables, five variables displayed type A evidence: emotion regulation ability, co-rumination, affective component of social cognition, and social skills; 4 variables showed type B: general coping, constructive coping, cognitive component of social cognition, and emotional intelligence; and 3 variables type C: language skills, academic competence, and autonomy in decision making.

**Table 2:** Skills and strengths variables.

**Variable**	**No. of studies (total No. of *r*)**	**Total sample size**	**ß [CI]**	**Heterogeneity (I^**2**^)**	**PRQ Measure Type^**i**^**	**Mean age (age range)**	**Type of evidence^**e**^**
**EMOTION REGULATION**							
**Emotion regulation ability**	**7 (41)**	**2,680**	0.30** [0.14–0.43]	**91.34**	**6 PQ 1 NQ**	**12.75**^**a**^ **(8–17)**	**A**
**Co-rumination**	**10 (19)**	**3,890**	0.33* [0.13–0.51]	**97.76**	**10 PQ 1 NQ**	**12.89**^**a**^ **(9–18)**	**A**
*Constructive coping and attributions*	*14 (43)*	*6,807*	0.25** [0.17–0.33]	*91.09*	*13 PQ 2 NQ*	*12.94^*a*^ (9–19)*	*B*
*General coping and attributions*	*3 (6)*	*1,384*	0.24* [0.04–0.42]	*91.16*	*1 PQ 2 NQ*	*11.49^*a*^ (8–15)*	*B*
**SOCIAL COGNITION**							
**Affective social cognition**	**5 (10)**	**1,843**	0.36** [0.10–0.57]	**97.16**	**5 PQ –**	**14.26 (11–18)**	**A**
**Prosocial motivation and sympathy**	**3 (5)**	**1,397**	0.31** [0.24–0.38]	**67.88**	**3 PQ –**	**13.22**^**a**^ **(11–18)**	**A**
*Cognitive social cognition*	*6 (15)*	*2,197*	0.27** [0.06–0.46]	*96.31*	*5 PQ 1 NQ*	*10.53^*a*^ (9–18)*	*B*
**SOCIAL SKILLS**							
**Social competence**	**26 (64)**	**11,167**	0.36** [0.29–0.44]	**95.51**	**17 PQ 9 NQ**	**12.8**^**a**^ **(8–18)**	**A**
**COGNITIVE AND LANGUAGE SKILLS**							
Language skills	2 (2)	419	0.18** [0.09–0.27]	0.00	1 PQ 1 NQ	12.73^a^ (11–16)	C
**ACADEMIC SKILLS**							
Academic competence	7 (12)	7,756	0.15* [0.02–0.28]	93.74	7 PQ –	13.29^a^ (8–18)	C
**EMOTIONAL INTELLIGENCE**							
*Emotional intelligence*	*3 (8)*	*814*	0.22** [0.09–0.34]	*14.59*	*1 PQ 2 NQ*	*14.37 (11–19)*	*B*
**AUTONOMOUS DECISION MAKING**							
Autonomy in decision-making	2 (3)	2,167	0.12* [0.00–0.24]	60.33	2 PQ –	13.78^a^ (10–18)	C

*NQ, Negative quality dimension (i.e., loneliness); PQ, positive quality dimension; PRQ, peer relationship quality; r, correlation coefficient; ß, estimated correlation coefficient; Bold, A evidence type; Italic, B evidence type. The PQ/NQ ratio depicts general patterns in data, however all coefficients were adapted and analyzed as positive dimensions*.

*^a^Mean age/age range suggest fair translatability to the context of early adolescence. ^i^Numbers do not fully correspond to the total numbers of studies due to multiple measures used in some studies. ^e^see Table 1 for detailed description of evidence types*.

*p < 0.05; **p < 0.01.

##### Identity

Within this theme, we identified three groups with 19 variables referring to (a) temperament, (b) relational dispositions, and (c) self-concepts (Table 3). Type A evidence in a positive direction was demonstrated for the following variables: hope and optimism (A^+^), sense of coherence (A^+^), adaptability, emotional stability, extraversion, and self-esteem; and in a negative direction for the shyness and rejection sensitivity variables. Type B was present for 7 variables (agreeableness, interpersonal goals, preference for solitude, trust beliefs, academic self-efficacy, social self-efficacy, and body image), D type for 4 variables (psychopathic traits, openness to experience, conscientiousness, and general self-efficacy). No C type evidence was present within this theme.

**Table 3:** Identity variables.

**Variable**	**No. of studies (total No. of *r*)**	**Total sample size**	**ß [CI]**	**Heterogeneity (I^**2**^)**	**PRQ Measure Type^**i**^**	**Mean age (age range)**	**Type of evidence^**e**^**
**TEMPERAMENTAL DISPOSITIONS**							
**Adaptability**	**2 (6)**	**2,293**	0.30** [0.22–0.38]	**0**	**1 PQ 2 NQ**	**15.42 (13–19)**	**A**
**Emotional stability**	**2 (2)**	**1,816**	0.34** [0.27–0.40]	**49.87**	**– 2 NQ**	**15.82 (13–19)**	**A**
**Extraversion**	**2 (2)**	**1,816**	0.46** [0.39–0.53]	**60.62**	**- 2 NQ**	**15.91 (12–19)**	**A**
*Agreeableness*	*3 (3)*	*244*	0.22** [0.18–0.26]	*7.02*	*1 PQ 1 NQ*	*12.07^*a*^ (10–14)*	*B*
Openness to experience	2 (2)	1,816	0.06 [−0.00–0.13]	38.01	– 2 NQ	15.82 (13–19)	D
Conscientiousness	2 (2)	1,816	−0.05 [−0.11–0.02]	37.45	– 2 NQ	15.91 (12–19)	D
Absence of psychopathic traits	3 (11)	960	0.02 [−0.16–0.20]	88.96	2 PQ 1 NQ	14.13 (10–18)	D
**RELATIONAL DISPOSITIONS**							
**Shyness**	**8 (10)**	5,735	−0.35** [−0.46 to −0.22]	**96.32**	**2 PQ 6 NQ**	**12.91**^**a**^ **(9–15)**	**A**
**Rejection sensitivity**	**8 (15)**	**2,783**	-0.34** [−0.42 to −0.25]	**81.66**	**4 PQ 6 NQ**	**12.46**^**a**^ **(9–17)**	**A**
*Preference for solitude*	*6 (9)*	*5,545*	−0.22** [−0.35 to −0.08]	*96.40*	*1 PQ 5 NQ*	*13.34^*a*^ (9–20)*	*B*
*Connection-oriented interpersonal goals*	*6 (13)*	*1,485*	0.23** [0.13–0.32]	*71.42*	*5 PQ 1 NQ*	*12.25^*a*^ (9–17)*	*B*
**SELF-CONCEPTS**							
**Beliefs and self-perceived abilities**							
**Hope and optimism**	**8 (10)**	6,029	0.43** [0.29–0.56]	**97.49**	4 PQ 5 NQ	12.39^a^ (9–16)	**A**^**+**^
**Sense of coherence**	**4 (8)**	2,539	0.49** [0.33–0.63]	**94.23**	1 PQ 3 NQ	11.21^a^ (9–15)	**A**^**+**^
*Trust beliefs*	*3 (4)*	*593*	0.26* [−0.00–0.49]	*86.041*	–* 3 NQ*	*11.36^*a*^ (9–11,97)*	*B*
*Social self-efficacy*	*5 (8)*	*2,037*	0.28** [0.10–0.45]	*91.51*	*5 PQ*	*12.61^*a*^ (9–16)*	*B*
*Academic self-efficacy*	*5 (5)*	*2,652*	0.22** [0.11–0.32]	*86.40*	*5 PQ –*	*12.20^*a*^ (8–17)*	*B*
General self-efficacy	2 (6)	534	0.12 [−0.01–0.25]	43.43	3 PQ –	13.64^a^ (11–19)	D
**Multidimensional self-concepts**							
**Self-esteem**	**44 (73)**	**31,905**	0.35** [0.30–0.39]	**95.40**	**27 PQ 23 NQ**	**12.90**^**a**^ **(7–19)**	**A**
*Body image*	*2 (5)*	*308*	0.23* [0.02–0.43]	*70.40*	*3 PQ –*	*15.05 (14–16)*	*B*

*NQ, Negative quality dimension (i.e., loneliness); PQ, positive quality dimension; PRQ, peer relationship quality; r, correlation coefficient; ß, estimated correlation coefficient; Bold, A evidence type; Italic, B evidence type. The PQ/NQ ratio depicts general patterns in data, however all coefficients were adapted and analyzed as positive dimensions*.

*^a^Mean age/age range suggest fair translatability to the context of early adolescence. ^i^Numbers do not fully correspond to the total numbers of studies due to multiple measures used in some studies. ^e^see Table 1 for detailed description of evidence types*.

*p < 0.05; **p < 0.01.

##### Behavior and Health

This large theme contains clinical and non-clinical health related variables referring to mental and physical health divided into 5 groups: (a) general clinical spectrum, (b) clinical spectrum in relational context, (c) multidimensional behavioral and emotional issues, (d) non-clinical behavioral spectrum, and (e) physical health (Table 4). Amongst the 19 distinct variables, 3 showed type A evidence i.e., depression, social anxiety, and self-disclosure. Type B was present for 2 variables (prosocial behavior and social withdrawal), type C for 9 variables (internalizing symptoms, anxiety, externalizing symptoms, risky and delinquent behavior, hyperactivity, aggression, multidimensional issues, problematic relational behavior, and sport participation), and type D for 3 (emotional symptoms, substance use, antisocial behavior).

**Table 4:** Behavior and health variables.

**Variable**	**No. of studies (total No. of *r*)**	**Total sample size**	**ß [CI]**	**Heterogeneity (I^**2**^)**	**PRQ Measure Type^**i**^**	**Mean age (age range)**	**Type of evidence^**e**^**
**GENERAL CLINICAL SPECTRUM**							
**Internalizing spectrum**							
**Depression**	**71 (141)**	**34,077**	−0.30** [−0.35 to −0.25]	**95.90**	**54 PQ 30 NQ**	**12.56**^**a**^ **(8–19)**	**A**
*Somatic symptoms*	*5 (11)*	*738*	−0.20** [−0.31 to −0.07]	*86.50*	*4 PQ 2 NQ*	*12.13^*a*^ (10–15)*	*B*
Anxiety	18 (33)	5,723	−0.13* [−0.23 to −0.03]	92.97	29 PQ 4 NQ	12.61^a^ (8–18)	C
Internalizing symptoms (AS)	24 (47)	10,005	−0.17** [−0.23 to −0.12]	87.15	19 PQ 4 NQ	12.86^a^ (8–18)	C
Emotional Symptoms	6 (9)	2,412	−0.13 [−0.25–0.01]	71.50	3 PQ 2 NQ	12.70^a^ (9–18)	D
**Externalizing spectrum**							
Externalizing symptoms (AS)	20 (44)	8,864	−0.17** [−0.22 to −0.12]	79.84	18 PQ 3 NQ	12.76^a^ (8–18)	C
Risky/delinquent behavior	9 (17)	2,377	−0.12** [−0.20 to −0.03]	83.48	8 PQ 3 NQ	13.00^a^ (10–18)	C
Hyperactivity	2 (5)	790	0.14** [0.10–0.20]	0	3 PQ 1 NQ	12.84^a^ (10–18)	C
Substance use	4 (4)	615	0.02 [−0.19–0.22]	79.25	5 PQ –	14.21 (12–18)	D
**RELATIONAL CLINICAL SPECTRUM**							
**Internalizing spectrum**							
**Social Anxiety**	**33 (73)**	**16,394**	−0.34** [−0.41to −0.27]	**95.40**	**22 PQ 20 NQ**	**12.10**^**a**^ **(7–18)**	**A**
*Social withdrawal*	*6 (8)*	*1,029*	−0.24** [−0.31 to −0.17]	15.01	*4 PQ 3 NQ*	*11.27^*a*^ (9–17)*	*B*
**Externalizing spectrum**							
Aggression	27 (73)	17,726	−0.13** [−0.18 to −0.08]	86.04	19 PQ 9 NQ	12.04^a^ (6–18)	C
Antisocial behavior	8 (8)	2,885	−0.01 [−0.20–0.19]	96.10	6 PQ 2 NQ	12.11^a^ (9–18)	D
**Multidimensional behavioral and emotional issues**							
Internalizing/externalizing symptoms (AS)	9 (17)	6,912	−0.19** [−0.25 to −0.13]	81.95	5 PQ 4 NQ	12.35^a^ (7–18)	C
**NON-CLINICAL SPECTRUM**							
**Self – disclosure**	**7 (37)**	**1,985**	0.40** [0.23–0.54]	**94.05**	**5 PQ 2 NQ**	**12.70**^**a**^ **(9–18)**	**A**^**+**^
*Prosocial behavior*	*14 (19)*	*6,576*	0.27** [0.15–0.38]	*96.00*	*12 PQ 3 NQ*	*12.40^*a*^ (7–17)*	*B*
Problematic relational behavior	7 (26)	2,876	−0.16** [−0.24to−0.09]	64.50	6 PQ 3 NQ	12.63^a^ (9–16)	C
**PHYSICAL HEALTH**							
Sport participation	5 (10)	5,854	0.12* [0.00–0.22]	89.00	4 PQ 1 NQ	12.19^a^ (8–18)	C

*AS, aggregated scores; NQ, Negative quality dimension (i.e., loneliness); PQ, positive quality dimension; PRQ, peer relationship quality; r, correlation coefficient; ß, estimated correlation coefficient; Bold, A evidence type; Italic, B evidence type. The PQ/NQ ratio depicts general patterns in data, however all coefficients were adapted and analyzed as positive dimensions*.

*^a^Mean age/age range suggest fair translatability to the context of early adolescence. ^i^Numbers do not fully correspond to the total numbers of studies due to multiple measures used in some studies. ^e^ see Table 1 for detailed description of evidence types*.

* p < 0.05; ** p < 0.01.

##### Affect and Well-Being

Within this theme, we distinguish between the three groups: (a) feelings and emotions specific for the relational context, (b) feelings and emotions that constitute core affect, and (c) general well-being (Table 5). Amongst 6 variables, happiness (A^+^), positive affect, and self-perceived quality of life demonstrated the A type of evidence. The remaining three variables displayed B (negative affect and multidimensional perceived stress) and D type (jealousy).

**Table 5:** Affect and well-being variables.

**Variable**	**No. of studies (total No. of *r*)**	**Total sample size**	**ß [CI]**	**Heterogeneity (I^**2**^)**	**PRQ Measure Type^**i**^**	**Mean age (age range)**	**Type of evidence^**e**^**
**CORE AFFECT**							
**Happiness**	**6 (8)**	**5,299**	0.40** [0.34–0.47]	**85.02**	**4 PQ 4 NQ**	**12.29**^**a**^ **(8.3–18)**	**A**^**+**^
**Positive affect**	**5 (6)**	**3,212**	0.31** [0.23–0.39]	**76.33**	**2 PQ 3 NQ**	**12.26**^**a**^ **(8–17)**	**A**
*Negative affect*	*8 (9)*	*7,189*	−0.26** [−0.37 to −0.15]	*93.75*	*4 PQ 4 NQ*	*13.05^*a*^ (8–18)*	*B*
**FEELINGS AND EMOTIONS IN RELATIONAL CONTEXT**							
Jealousy	3 (8)	603	−0.10 [−0.39–0.21]	83.81	3 PQ –	11.51^a^ (9–15)	D
**GENERAL WELL-BEING**							
**Perceived QoL and satisfaction**	**23 (34)**	**27,395**	0.39** [0.35–0.43]	**93.424**	**15 PQ 11 NQ**	**13.32**^**a**^ **(7–19)**	**A**
*Multidimensional perceived stress*	*8 (11)*	*4,644*	−0.20** [−0.33 to −0.08]	*94.656*	*8 PQ 1 NQ*	*14.27 (10–19)*	*B*

*NQ, Negative quality dimension (i.e., loneliness); PQ, positive quality dimension; PRQ, peer relationship quality; r, correlation coefficient; ß, estimated correlation coefficient; Bold, A evidence type; Italic, B evidence type. The PQ/NQ ratio depicts general patterns in data, however all coefficients were adapted and analyzed as positive dimensions*.

*^a^Mean age/age range suggest fair translatability to the context of early adolescence. ^i^Numbers do not fully correspond to the total numbers of studies due to multiple measures used in some studies. ^e^ see Table 1 for detailed description of evidence types*.

*p < 0.05; **p < 0.01.

#### Environmental Cluster

##### Family

The family theme included 14 variables constituting 4 groups: (a) family unit, (b) relationships between family members, (c) parental factors, and (d) childhood maltreatment (Table 6). Amongst these 14 variables, only parental support showed type A evidence, secure attachment to parents showed type B. Most variables demonstrated type C (family climate, child perception of interparental conflict, sibling relationship quality, parenting variables, and childhood abuse and neglect) and type D evidence (parent child communication, parental interpersonal skills, economic factors, parental perceptions of interparental conflict, parental mental illness, and exposure to family violence).

**Table 6:** Family variables.

**Variable**	**No. of studies (Total No. of *r*)**	**Total sample size**	**ß [CI]**	**Heterogeneity (I^**2**^)**	**PRQ Measure Type^**i**^**	**Mean age (age range)**	**Type of evidence^**e**^**
**FAMILY UNIT FUNCTIONING**							
Family climate	5 (10)	4,520	0.19** [0.08–0.29]	92.00	3 PQ 3 NQ	12.14^a^ (9.5–18)	C
Economic factors	6 (6)	10,950	0.09 [0.03–0.15]	87.30	3PQ 3 NQ	11.01^a^ (7–14)	D
**RELATIONSHIPS BETWEEN FAMILY MEMBERS**							
*Parental Support*	*25 (43)*	*9,923*	0.29** [0.23–0.36]	*93.01*	*23 PQ 2 NQ*	*12.69^*a*^ (8–19)*	*B*
*Secure attachment to parents*	*38 (174)*	*27,862*	0.24** [0.21–0.28]	*88.35*	*42 PQ 1 NQ*	*13.09^*a*^ (8–18)*	*B*
Functional parent child communication	3 (21)	1,388	0.18 [−0.04–0.38]	91.46	2 PQ 2 NQ	13.72^a^ (10–18)	D
Inter-parental conflict – CP	5 (41)	1,026	−0.14** [−0.21 to −0.08]	40.37	5 PQ 1 NQ	12.43^a^ (8–16)	C
Sibling relationship	4 (17)	1,520	0.17** [0.14–0.20]	0	5 PQ 1 NQ	11.83^a^ (8–17)	C
Inter-parental conflict – PP	4 (67)	957	−0.04 [−0.07 0.00]	0	3 PQ 1 NQ	11.89^a^ (8–15)	D
**PARENTAL FACTORS**							
*Positive parenting*	*16 (39)*	*12,282*	0.20** [0.10–0.30]	*96.38*	*15 PQ 7 PQ*	*11.73^*a*^ (6–18)*	*B*
Negative parenting	18 (52)	11,733	−0.13** [−0.07 to −0.19]	88.30	14 PQ 5 NQ	11.90^a^ (6–18)	C
Parental interpersonal skills	2 (6)	241	0.19 [−0.10 to −0.45]	76.80	2 PQ –	14.37 (12–16)	D
Parental mental illness	3 (7)	337	−0.09* [−0.17 to −0.02]	0	2 PQ 1 NQ	11.38^a^ (7–17)	D
**CHILDHOOD MALTREATMENT**							
Childhood abuse and neglect	3 (10)	3,557	−0.14* [−0.27 to 0.01]	92.45	3 PQ –	12.25^a^ (8–17.5)	C
Exposure to family violence	2 (2)	1,284	−0.08** [−0.13 to −0.02]	0	2 PQ –	12.5^a^ (11–15)	D

*CP, child perception; NQ, Negative quality dimension (i.e., loneliness); PP, parental perception; PQ, positive quality dimension; PRQ, peer relationship quality; r, correlation coefficient; ß, estimated correlation coefficient; Bold, A evidence type; Italic, B evidence type. The PQ/NQ ratio depicts general patterns in data, however all coefficients were adapted and analyzed as positive dimensions*.

*^a^Mean age/age range suggest fair translatability to the context of early adolescence. ^i^Numbers do not fully correspond to the total numbers of studies due to multiple measures used in some studies. ^e^see Table 1 for detailed description of evidence types*.

*p < 0.05; **p < 0.01.

##### Peer Group

This small theme contained 4 variables referring to (a) peer group characteristics and (b) experiences of an individual within the peer group (Table 7). Peer group inclusion shows type A evidence, victimization and peer problems type B, and peer deviance type C.

**Table 7:** Peer group variables.

**Variable**	**No. of studies (total No. of *r*)**	**Total sample size**	**ß [CI]**	**Heterogeneity (I^**2**^)**	**PRQ Measure Type^**i**^**	**Mean age (age range)**	**Type of evidence^**e**^**
**EXPERIENCES IN THE PEER GROUP**							
**Inclusion**	**17 (25)**	**13,819**	0.38** [0.27–0.48]	**97.42**	**11 PQ 7 NQ**	**12.49**^**a**^ **(7–18)**	**A**
*Victimization*	*41 (105)*	*23,228*	−0.26** [−0.32 to −0.21]	*94.61*	*26 PQ 13 NQ*	*12.40^*a*^ (8–17)*	*B*
*Peer related stress*	*6 (7)*	*3,182*	−0.27** [−0.36 to −0.18]	*71.60*	*4 PQ 2 NQ*	*11.82^*a*^ (9–18)*	*B*
**PEER GROUP CHARACTERISTICS**							
Peers' deviant behavior	6 (14)	4,936	−0.12* [−0.23 to−0.01]	92.62	6 PQ –	13.73^a^ (10–18)	C

*NQ, Negative quality dimension (i.e., loneliness); PQ, positive quality dimension; PRQ, peer relationship quality; r, correlation coefficient; ß, estimated correlation coefficient; **Bold**, A evidence type; Italic, B evidence type. The PQ/NQ ratio depicts general patterns in data, however all coefficients were adapted and analyzed as positive dimensions*.

*^a^Mean age/age range suggest fair translatability to the context of early adolescence. ^i^Numbers do not fully correspond to the total numbers of studies due to multiple measures used in some studies. ^e^see Table 1 for detailed description of evidence types*.

*p < 0.05; **p < 0.01.

##### School

The school theme contained 9 variables in three groups, related to (a) academic performance, (b) experiences in the school setting, and (c) relationship with the teacher (Table 8). Our results show: type A evidence for positive attitudes toward school and school belonging; type B for student-teacher relationship and academic engagement; type C for academic performance, mastery motivation and school adjustment; and type D for the classroom autonomy variable.

**Table 8:** School variables.

**Variable**	**No. of studies (total No. of *r*)**	**Total sample size**	**ß [CI]**	**Heterogeneity (I^**2**^)**	**PRQ Measure Type^**i**^**	**Mean age (age range)**	**Type of evidence^**e**^**
**INDIVIDUAL ACADEMIC RELATED FACTORS**							
**Positive attitudes toward school**	**5 (9)**	**3,840**	0.34** [0.16–0.49]	**96.10**	**5 PQ –**	**13.04**^**a**^ **(10–19)**	**A**
*Academic engagement*	*15 (30)*	*12,304*	0.23** [0.18–0.27]	*75.49*	*13 PQ 7 NQ*	*12.00^*a*^ (8–18)*	*B*
Academic performance	16 (29)	7,770	0.10** [0.06–0.15]	71.44	15 PQ 5 NQ	11.18^a^ (7–16)	C
Mastery motivation	7 (17)	3,531	0.15** [0.10–0.19]	46.52	9 PQ –	13.33^a^ (11–17)	C
School adjustment	3 (10)	2,508	0.16** [0.05–0.27]	0.01	2 PQ 2 NQ	12.29^a^ (10–16)	C
**EXPERIENCES IN THE SCHOOL SETTING**							
**School belonging**	**14 (20)**	**9,662**	0.31** [0.26–0.37]	**88.13**	**10 PQ 4 NQ**	**12.68**^**a**^ **(8–18)**	**A**
**PE for academic engagement**	**3 (5)**	**1,009**	0.33** [0.25–0.41]	**48.05**	**3 PQ –**	**12.34**^**a**^ **(10–16)**	**A**
Autonomy in classroom	2 (2)	1,586	0.55 [−0.32–0.92]	99.72	2 PQ –	12.41^a^ (11–17)	D
**RELATIONSHIP WITH TEACHER**							
*Relationship with Teacher*	*19 (47)*	*15,028*	0.27** [0.21–0.33]	*93.38*	*17 PQ 3 NQ*	*12.23^*a*^ (8–17.5)*	*B*

*NQ, Negative quality dimension (i.e., loneliness); PQ, positive quality dimension; PRQ, peer relationship quality; PE, peers' expectations; r, correlation coefficient; ß, estimated correlation coefficient; Bold, A evidence type; Italic, B evidence type. The PQ/NQ ratio depicts general patterns in data, however all coefficients were adapted and analyzed as positive dimensions*.

*^a^Mean age/age range suggest fair translatability to the context of early adolescence. ^i^Numbers do not fully correspond to the total numbers of studies due to multiple measures used in some studies. ^e^see Table 1 for detailed description of evidence types*.

*p < 0.05; **p < 0.01.

##### Internet and Technology

Within this theme, we identified 7 variables in four groups referring to (a) usage tendencies, (b) cyberbullying, (c) online communication, and (d) online activities (Table 9). Problematic technology use showed type A; quality of online communication and online victimization showed type C; time spent online, online aggression, communication with strangers, and online information seeking displayed type D.

**Table 9:** Internet and technology variables.

**Variable**	**No. of studies (total No. of *r*)**	**Total sample size**	**ß [CI]**	**Heterogeneity (I^**2**^)**	**PRQ Measure Type^**i**^**	**Mean age (age range)**	**Type of evidence^**e**^**
**GENERAL USAGE TENDENCIES**							
**Problematic/addictive IandT use**	**7 (12)**	**10,541**	−0.32** [−0.44 to −0.19]	**97.33**	**3 PQ 5 NQ**	**15.05 (10–19)**	**A**
Time spent online	7 (11)	5,506	−0.07 [−0.16–0.01]	89.28	4 PQ 4 NQ	14.14 (12–19)	D
**CYBERBULLYING**							
Online victimization	8 (12)	4,231	−0.14** [−0.20 to −0.93]	59.95	4 PQ 4 NQ	14.2 (10–18)	C
Online aggression	4 (6)	1,777	−0.12 [−0.32 to 0.09]	93.32	2 PQ 2 NQ	14.4 (11–18)	D
**ONLINE COMMUNICATION**							
Quality of communication	3 (7)	2,159	0.18** [0.07–0.28]	76.36	3 PQ 1 NQ	13.04^a^ (10–17)	C
Communication with strangers	2 (3)	1,782	−0.15 [−0.32–0.03]	93.02	2 PQ 1 NQ	13.1^a^ (10–17)	D
**TYPE OF ONLINE ACTIVITIES**							
Information seeking	2 (3)	2,038	0.02 [−0.20 to −0.25]	95.73	2 NQ 1 PQ	11.27^a^ (8–16)	D

*IandT, Internet and technology; NQ, Negative quality dimension (i.e., loneliness); PQ, positive quality dimension; PRQ, peer relationship quality; r, correlation coefficient; ß, estimated correlation coefficient; Bold, A evidence type; Italic, B evidence type. The PQ/NQ ratio depicts general patterns in data, however all coefficients were adapted and analyzed as positive dimensions*.

*^a^Mean age/age range suggest fair translatability to the context of early adolescence. ^i^Numbers do not fully correspond to the total numbers of studies due to multiple measures used in some studies. ^e^see Table 1 for detailed description of evidence types*.

*p < 0.05; **p < 0.01.

##### Community

This theme comprises 4 variables clustered into two groups, referring to (a) socio-demographic factors and (b) experiences of community setting (Table 10). Our results showed the A type of evidence for the variable relationships with non-parental adults (A+), B type for community connectedness, and D type for exposure to community violence and economic factors.

**Table 10:** Community variables.

**Variable**	**No. of studies (total No. of *r*)**	**Total sample size**	**ß [CI]**	**Heterogeneity (I^**2**^)**	**PRQ Measure Type^**i**^**	**Mean age (age range)**	**Type of evidence^**e**^**
**EXPERIENCES IN THE COMMUNITY SETTING**							
**Relationships with significant others**	**3 (3)**	**2,114**	0.66** [0.46–0.79]	**96.44**	**3 PQ –**	**14.93 (14.1–15.9)**	**A**^**+**^
*Community connectedness*	*5 (8)*	*11,137*	0.24** [0.19−0.2*9]*	*84.43*	*5 PQ –*	*12.89^*a*^ (9–19)*	*B*
Exposure to community violence	4 (9)	2,148	−0.10* [−0.16 to −0.03]	13.23	4 PQ –	13.00^a^ (10–17)	D
**SOCIO-DEMOGRAPHIC FACTORS**							
Economic factors	2 (3)	2,194	−0.07** [−0.11 to −0.03]	0	2 PQ –	13.88^a^ (10–19)	D

*NQ, Negative quality dimension (i.e., loneliness); PQ, positive quality dimension; PRQ, peer relationship quality; r, correlation coefficient; ß, estimated correlation coefficient; Bold, A evidence type; Italic, B evidence type. The PQ/NQ ratio depicts general patterns in data, however all coefficients were adapted and analyzed as positive dimensions*.

*^a^Mean age/age range suggest fair translatability to the context of early adolescence. ^i^Numbers do not fully correspond to the total numbers of studies due to multiple measures used in some studies. ^e^see Table 1 for detailed description of evidence types*.

*p < 0.05; **p < 0.01.

#### Hierarchical Maps of Main Findings

**Figure 3:**
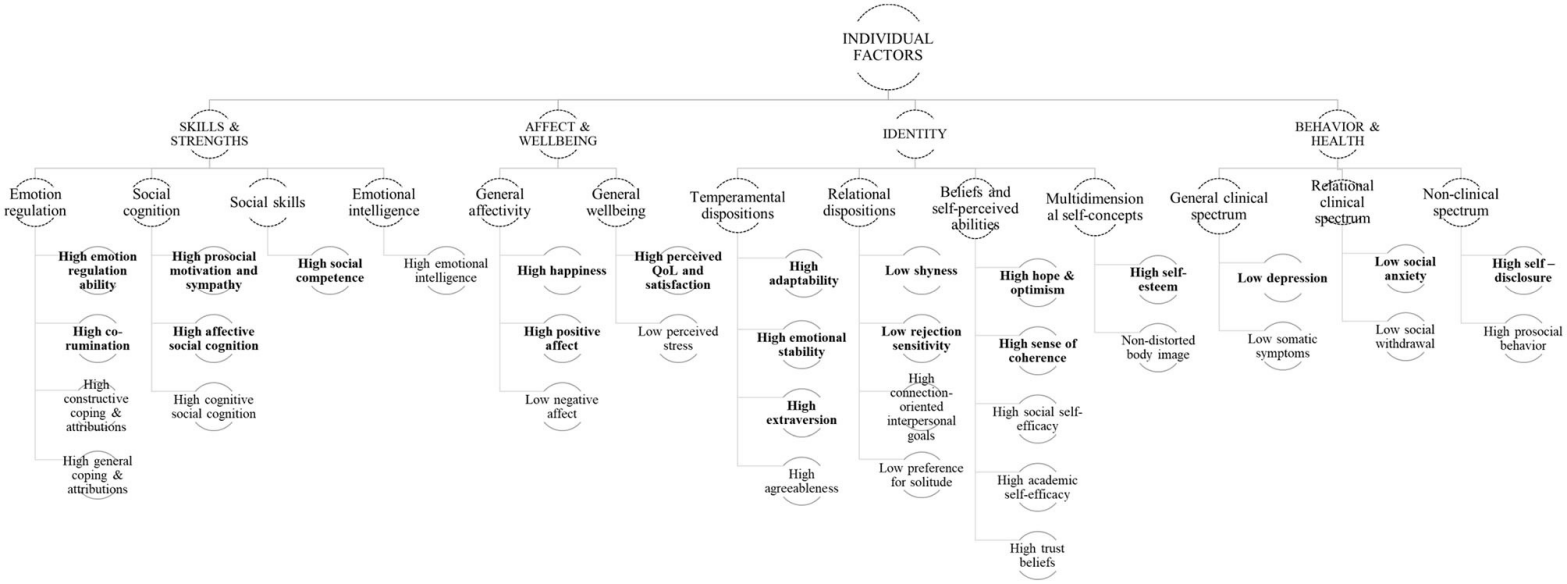
Hierarchical map of individual factors. The variables were named to reflect the positive association with the high PRQ. **- - -** qualitaitively derived constructs; __ quantitative evidence; bold – A/A^(+)^ evidence type; BLOCK CAPTALS – clusters and themes.

**Figure 4:**
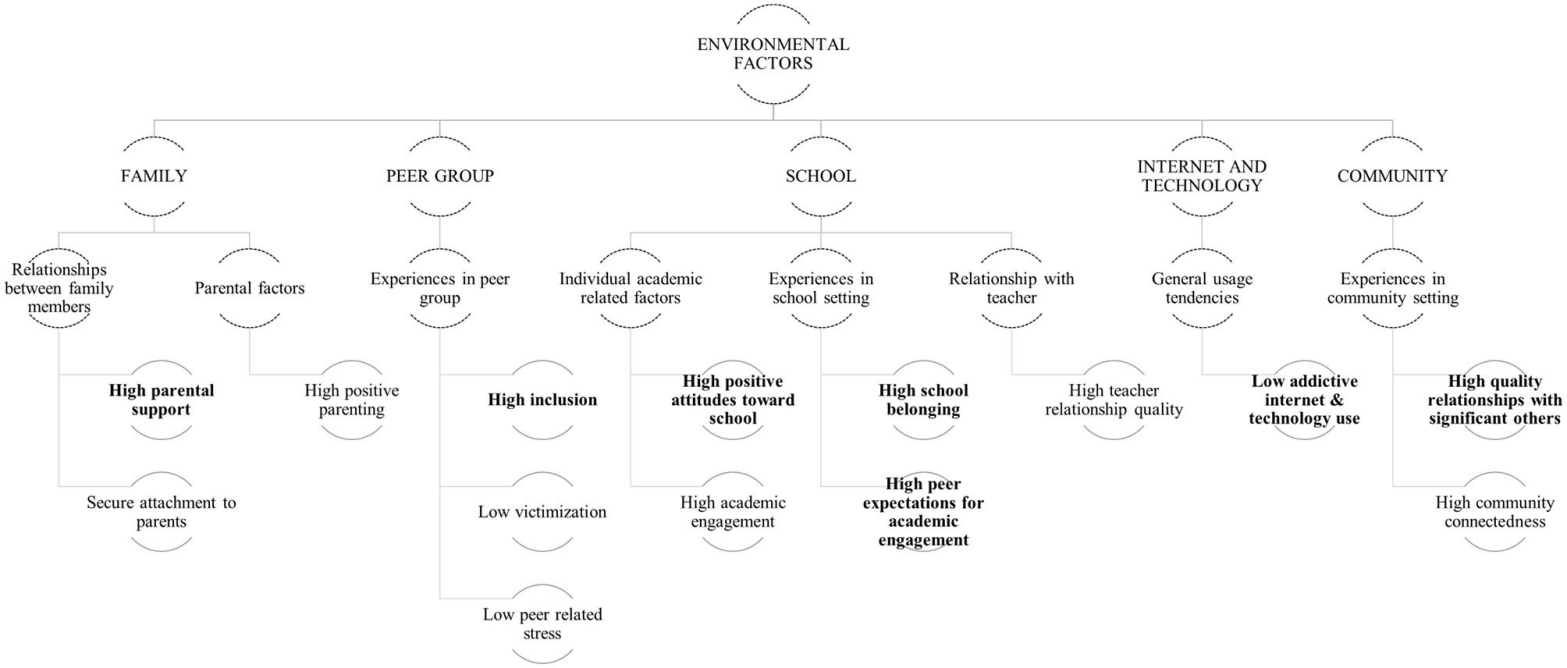
Hierarchical map of environmental factors. The variables were named to reflect the positive association with the high PRQ. **- - -** qualitaitively derived constructs; __ quantitative evidence; bold – A/A^(+)^ evidence type; BLOCK CAPTALS – culsters and themes.

As described in the methods section, salient findings were used to form the hierarchical maps of evidence and to facilitate the development of the integrated model in the mixed methods synthesis. The two maps were created based on the two clusters of potential determinants i.e., individual (Figure 3) and environmental (Figure 4) cluster.

### Discussion

This is the first review to systematically examine and quantify the existing evidence on potential determinants of SPR in early adolescents, where SPR are defined in terms of PRQ. Following a comprehensive systematic literature review, data were analyzed in three stages, i.e., first, qualitative clustering of factors into meaningful and internally comparable groups of potential determinants, second, exploratory meta-analysis to inform on the relative strength of associations with PRQ, and third, mixed methods synthesis where we apply established psychological theory to our findings to propose an integrated model of potential determinants of early adolescent SPR, which can serve as a foundation for future research.

### Integrated Model of Supportive Peer Relationships

**Figure 5:**
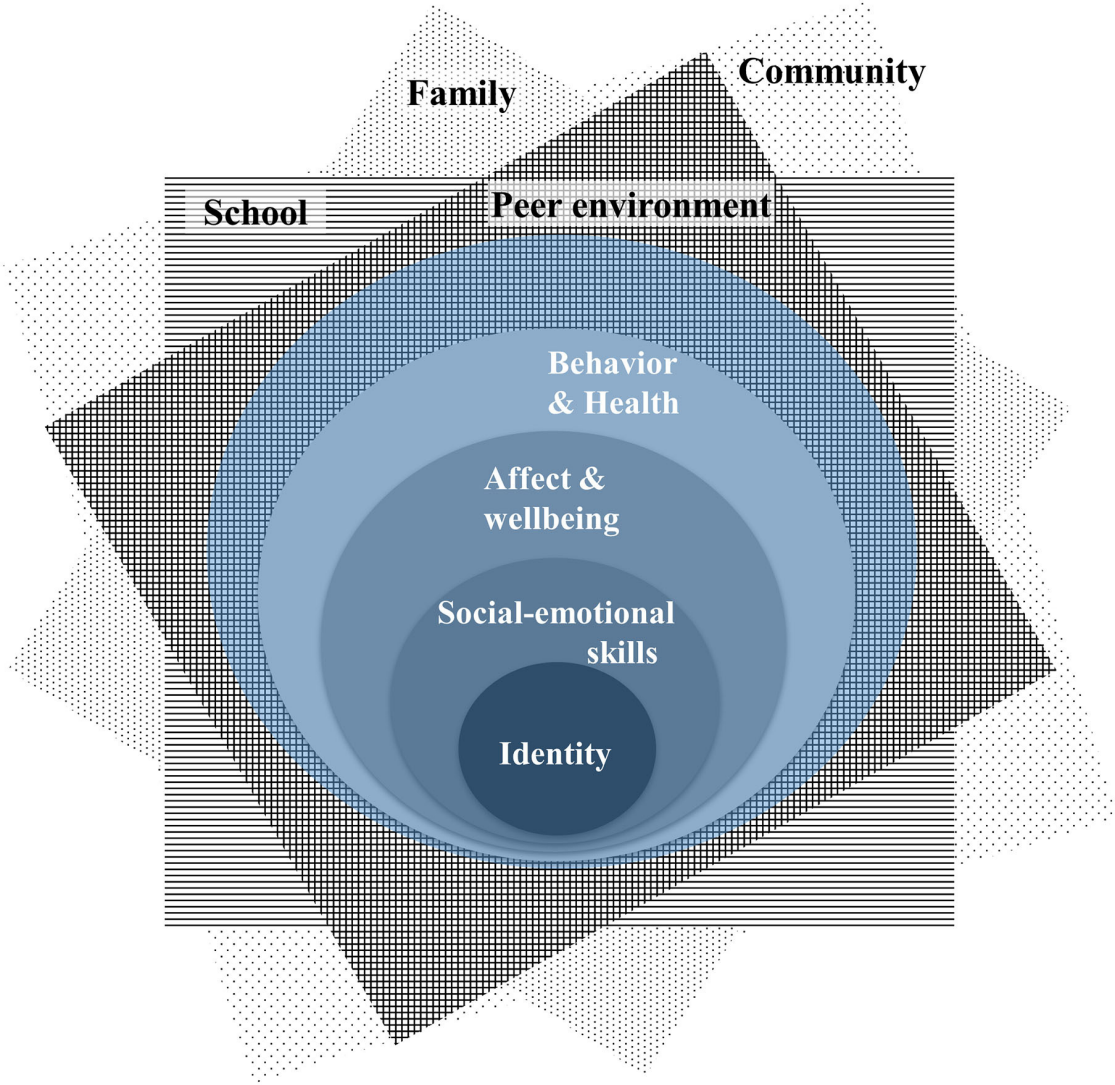
Integrated model of supportive peer relationships in early adolescence. Black letters/rectangles, environmental planes; white letters/circles, self-building-blocks. Identity and social-emotional skills, core self-building blocks; affect and well-being, bridging self-building-block; behavior and health, peripheral self-building-block. Intensity of shading/pattering indicates higher relevance of a block/plane for the SPR. Transparent colored circle represents a self-perceived circle of SPR as an intersection between the self and the peer environmental plane.

Our model (Figure 5) encompasses key individual and environmental factors, as determined based on the strength of the associations identified in our meta-analyses. Our main findings underscore individual factors related to identity and social–emotional skills as the most potent targets for future empirical research in the field. Next, we emphasize the significance of the school environment for peer interactions in this age group. Finally, we identify an alarming gap in research on the influence of the virtual or online environment on SPR given its undeniable importance as a globally expanding social interaction setting. The model can serve as an organizational framework for integration of diverse findings on SPR, including those based on quantitative and qualitative data. The model depicts individual factors as self-building-blocks, which interact with environmental factors represented as planes as part of an integrated system. SPR are constituted by continuous reciprocal interactions between the self and the peer environment and modified by continuous influences from the surrounding environmental planes (school, community, and family; Magnusson and Håkan, 2007).

#### Self-Building-Blocks

Here, the self can be defined as a set of psychological processes that allow self-reflection, affect the nature of conscious experience, underlie all perceptions, beliefs, and feelings about oneself and allow deliberate regulation of one's own behavior (Leary and Tangney, 2011). Hence, self-building-blocks are specific internal functions that constitute an individual's sense of self and agency within the environment. And the interaction between self-building blocks and environmental planes can be conscious and/or subconscious.

The self-building-blocks are only abstractions covering different aspects of an organism (self) that functions as an organized whole (Magnusson and Håkan, 2007), so they cannot be fully represented in a linear fashion. However, for didactical reasons, we propose that the inner structure of the self originates from the core self-building-blocks (identity and social-emotional skills), which interact with an individual's affective response (i.e., bridging self-building-block) that further shapes the experience of the surrounding environment and the behavioral response (i.e., peripheral self-building-block). Hence, within the proposed inner structure the core self-building-blocks have a processing and regulatory function. The bridging self-building-block allows internal communication (defined as a means of sending and/or receiving information) between the core and peripheral block. Whereas, the peripheral self-building-block represents the self, interacting with a specific environmental plane (e.g., interaction with a best friend in school context or with a younger sibling in family context) and as such has a communicative function between the self and the environment.

Our results show a constant moderate to strong association between PRQ and all aspects of identity, social-emotional skills set, core affect, well-being, internalizing, and prosocial behaviors and provide initial evidence for the interdependence between the developmental pathways of self-building-blocks and SPR and for the reciprocal interactions between the self and the peer environment. This association is especially convincing for the core self-building-blocks. Yet, the specific functions of each self-building-block and dynamic interaction between them that results in a holistic response of the self are yet to be understood. Further, the mechanisms of the interaction between the self and the environmental planes and the intersecting role of SPR in these processes are still mainly unknown. These are all important avenues for future research.

#### Environmental Planes

The concept of environmental planes is based on the alternative “networked” adaption of the Bronfenbrenner's ecological systems theory model by Neal and Neal (2013) that views ecological systems as an overlapping arrangement of structures, each directly or indirectly connected to the others by the direct and indirect social interactions of their participants. We focus on the quality of these interactions (relationships), hence in our model, each environmental plane encompasses setting-specific interpersonal relationships, group dynamics, hierarchy and other sociopolitical factors (e.g., socio-economic status, culture etc.) that influence relationships in the current context. The planes are in constant reciprocal interactions with each other, with elements from other planes and with the self-building blocks via shred social ties. Additionally, environmental planes are influenced by the behavioral response of the self, yet this influence may vary from macroscopically unnoticeable to extremely noticeable (Brown and Larson, 2009) depending on a context, type of interaction, number of interacting elements etc.

The salient associations between SPR and at least one variable related to the setting specific relationships (i.e., parental attachment, teacher relationship, relationships with significant others, peer group inclusion) in each of the environmental planes provides an initial evidence for the socially mediated interdependence and reciprocal interactions between the different environmental planes and their influence on SPR. The salient associations with context specific variables (e.g., school belonging; academic engagement etc.) indicates the significance of sociopolitical factors (e.g., influences of educational policies) within the planes. Yet, elaborated understanding of the interpersonal and sociopolitical factors that influence SPR in each environmental plane is missing and the interpersonally mediated mechanisms of environmental influences on SPR are still unknown. Future research should focus on further exploration of these processes.

#### A Hypothetical Example of Supportive Peer Relationship in the School Context

The following example illustrates how our framework can be used to integrate diverse factors to understand SPR in a certain environmental context. Early adolescents experience their friendships based on the holistic response of the self in a certain environmental context, where affect, cognition and corresponding behaviors are defined by initial responses of core self-building-blocks. The core building-blocks themselves are shaped by previous and modified by current relational experiences with family members (family plane), peers (peer plane), and non-parental adults (community and school plane; Davidson and McEwen, 2012; Wong et al., 2018). Every model component is to a certain extent defined by these reciprocal relationships (Brown and Larson, 2009), as exemplified by the interaction between the self—e.g., with an empathic but no-non-sense identity—and a friend in the classroom environment. Here, in a direct interaction with a friend the self will display affective and behavioral relational patterns (e.g., variable shyness evidence type A) shaped by previous relational experiences, which influence the self-interpreted quality of the current dyadic interaction (e.g., Ditommaso et al., 2003; Erskine, 2018). Thus, abruptness from the friend may reduce the perceived quality of the interaction if abruptness has been experienced previously in a negative interaction in the classroom (e.g., experienced victimization evidence type B). However, if the current interaction shows a new pattern, previously unknown to the self (e.g., friend apologizing for being abrupt and explaining it was driven by their own anxiety—connection oriented interpersonal goals—type B), that new pattern may be internalized in line with the self-identity (i.e., empathizing with friend's anxiety when they are abrupt—affective social cognition—type A; prosocial motivation and sympathy—type A) and—if reinforced—applied to other relationships or contexts. Alternatively, if it is insufficiently repeated in future interactions, it can be overwritten by a previously established pattern (i.e., abruptness indicates rudeness and is negative—rejection sensitivity—type A). Thus, the integrated self-building-block's response in repeated dyadic interactions may act as a self-reinforcing function with the aim to sustain the known or to establish a new relational pattern (Erskine, 2018).

It is not possible to name all factors that determine the direction of the self-reinforcing response, yet we hypothesize an important role of the surrounding environmental planes (interpersonal and sociopolitical factors) in this process. The friendship dyad is a part of the larger peer environmental plane, which is here embedded in the school environmental plane. Hence, the friendship dyadic interaction is further shaped by the surrounding peer environment (e.g., peer group dynamics and hierarchy—peer related stress—type B) and by the school environment (e.g., positive attitudes toward school—type A). The less observable influence in the opposite direction (i.e., the influence of the dyad on the classroom environment) is also part of the system (Brown and Larson, 2009), yet we were unable to capture it based on available data. Finally, in addition to the described direct influences on the formation of the core-building blocks, family and community planes may directly influence the friendship dyad and/or classroom environment (e.g., by providing resources for meaningful and fun interaction—positive parenting—type B; community connectedness—type B).

All described processes over time result in more or less stable routinely displayed interaction patterns (e.g., supporting each other, teasing, sharing secrets etc.) within the friendship dyad. They also determine the hierarchical positioning of each dyad member (e.g., inclusion—type A) and the dyad itself within the classroom system. However, alternative patterns and behaviors (e.g., social anxiety—type A) may emerge due to chronic exposure to adversity, acute extremely stressful experiences or in situations that resemble past traumatic experiences (e.g., family violence, bullying, a new environment during the school transition etc.; D'Andrea et al., 2012; Alisic et al., 2014; Cook et al., 2017). The wholeness of all described relational interactions and corresponding responses of the self-building-blocks within the model constitute the “personal lens” through which the self-experiences its social environment (Kestly, 2016). This personal lens may be operationalized as social-emotional well-being (e.g., quality of life—type A). Hence, mechanisms of establishment and malleability of these relational patterns are critical for the long-term well-being.

Bellow, we offer empirically based speculations on significance of SPR for well-being in early adolescence and vice versa by discussing a role of each model component (i.e., self-building-block and environmental plane) supported by the evidence from our study and from the extant literature. The speculations and the hypothetical example are based on Magnusson's functional interaction principles (i.e., reciprocity, interdependence, and non-linearity) and on the literature based assumption supported by our empirical evidence (happiness—type A^+^; perceived quality of life—type A) that the lack of SPR is inherently interlinked to low social-emotional well-being.

### Identity as a Core Self-Building-Block

We place identity at the center of our model due to the strength of the associations demonstrated between SPR and identity-related variables. This is in line with previous research (Lee and Robbins, 1998; Stets and Burke, 2000; Karcher et al., 2008), which emphasizes the significance of identity for PR and well-being. According to our findings, identity encompasses temperamental dispositions, self-concepts, and relational dispositions, defined as relatively well-established trait-like functional, emotional and cognitive patterns, developed and reshaped through continuous reciprocal interaction between an individual and the environment (Kunnen and Bosma, 2003).

Extant literature suggests that early experiences with caregivers are crucial for the development of general identity aspects (such as general self-concepts and temperament), whereas domain specific aspects such as self-efficacy are more influenced by “out of family” social relations (Orth and Robins, 2019). Again, this supports the reciprocal interactions we propose in our model between core blocks and environmental planes, which result in a self that is continually shaped and re-shaped by interactions with the environment.

Indeed, supporting the reciprocal nature of a core role of identity in SPR, such relationships might serve to attenuate developmental trajectories of some personality aspects (e.g., Reitz et al., 2014; Chen et al., 2019). Low self-esteem (Sowislo and Orth, 2013), rejection sensitivity (Romero-Canyas et al., 2010; Liu et al., 2014), shyness and preference for solitude are found to be vulnerability factors for depression in youth and adults (Hay et al., 2004; Gazelle et al., 2014). On the other hand, hope, optimism, sense of coherence, and self-efficacy might be potent resilient factors in humans of all ages (e.g., Gorrese and Ruggieri, 2013; Schiavon et al., 2016; Alimujiang et al., 2019). Hence, identity determines SPR and well-being, is simultaneously shaped by the same SPR, and this dynamism may lead to a change in well-being and potentially in some identity aspects over time.

Despite such an important role of identity in SPR and vice versa, the research on identity developmental trajectories in children and early adolescents is mainly focused on self-esteem and is fragmented and somewhat conflicting (Orth and Robins, 2019). Future research should focus on mapping potential key timings and key contexts in developmental trajectories especially of under-researched protective traits (i.e., hope, optimism, sense of coherence, self-efficacy). Besides exploring the levels (i.e., high vs. low) of individual identity aspects, other dimensions such as fluctuations over time (Orth and Robins, 2019) and interrelatedness between them need to be addressed in order to move toward a comprehensive understanding of human identity and the interdependence between identity and interpersonal relationships (including SPR). Finally, a role of sociopolitical factors within environmental developmental contexts remains mainly unrecognized (e.g., Lardier et al., 2019; Opara et al., 2020) and hence the knowledge on their influence on the developmental pathways of human identity remains limited (e.g., Zeldin et al., 2017; Vézina and Poulin, 2019).

### Social-Emotional Skills as a Core Self-Building-Block

Social-emotional skills i.e., abilities necessary to understand and manage emotions, set and achieve positive goals, feel, and show empathy for others, establish and maintain positive relationships, and make responsible decisions (adapted from Casel, 2013) are the second central component of our model. In fact, identity and social-emotional skills can be understood as the two sides of the same coin (i.e., core self-building-blocks) that develop and act together in order to orchestrate the holistic response of the self in a certain environmental context (e.g., Calkins and Fox, 2002). They comprise multidimensional and partially overlapping constructs (Abrahams et al., 2019) emotion regulation, social cognition, social skills, and emotional intelligence that showed a consistent salient association with SPR in our analysis. Affect underpins all aspects of cognition; hence, these skills are key factors for the sense of self (Gross, 2013a) and for understanding and interpreting the environment (Immordino-Yang, 2015). This association is reciprocal—the affective expression of an individual influences how others perceive them (Kim and Son, 2015) and the core blocks are the main regulator in this process.

In line with the reciprocal interactions that shape shared developmental contexts we are proposing in our model, neuroscientific evidence has suggested the importance of the family (i.e., early experiences with caregiver; Shaver and Mikulincer, 2007) and the social environment (i.e., peer relationships) in the period of early adolescence for the development of these skills (Lamblin et al., 2017; Wong et al., 2018). Yet, this is the first meta-analysis to provide evidence on the relevance of emotion regulation and social skills for SPR in typically developing youth. In previous work, a significant association between SPR and social cognition skills has been demonstrated in preschool and early school populations (2–10 years; Slaughter et al., 2015). Yet, our findings suggest a stronger association in early adolescence, which further underscores the significance of this particular developmental stage.

The role of social-emotional skills in SPR highlighted here is consistent with a more general role of such skills for well-being, which has been previously linked to their self-regulatory function and impact on mental health outcomes (Kim et al., 2013; Germine et al., 2015; Von Salisch, 2018). Hence, an important task for future research is to examine the associations between the core self-building-blocks (i.e., identity and skills), SPR, and general well-being (i.e., bridging and peripheral blocks) together thus we can begin to parcel out the role SPR have in allowing these aspects of self to promote well-being and specific metal health outcomes.

Co-rumination is an important constituent of the social-emotional skills set as conceptualized in our model. Given the widely demonstrated maladaptive role of rumination in the context of mental health outcomes (Flett et al., 2016), the importance of co-rumination (evidence type A) in SPR is particularly interesting due to its positive direction. Our results are consistent with a growing literature demonstrating the heterogeneity of rumination sub-constructs in terms of their roles in psychological well-being. For example, Burns et al. (2019) demonstrate a more adaptive role of reflection than of brooding rumination sub-components in terms of mental health outcomes. Further, observational findings by Rose et al. (2014) suggest that certain aspects of co-rumination (i.e., extensively talking, rehashing problems and mutual encouragement, which in other research are positively associated with each other; Felton et al., 2019) are associated with positive friendship quality, whereas other aspects (i.e., dwelling on negative affect) do not show this association. Hence, the “positive” aspects of co-rumination can create closeness through sharing, perception of a potential common goal, the prospect of successfully overcoming problems and the experience of mutual support. These findings contribute to a better understanding of early adolescents' social realm and have a therapeutic value, especially for peer support programs. However, the Rose et al. (2014) also suggested links with depression (peripheral self-building-block) that again indicates the general need for integrated understanding of interactions between the different self-building-blocks, and SPR in different environmental contexts that would allow researchers to understand if/how the positive aspects of co-rumination can be harnessed as a meaningful resource for future interventions.

### Affect and Well-Being as a Bridging Self-Building-Block

Core affect (i.e., consciously accessible elemental processes of pleasure and activation; Russell and Barrett, 1999) and general well-being (i.e., inter- and intraindividual levels of positive functioning; Burns, 2015) are important constituents of our integrated model due to strong associations of identified related variables (i.e., happiness, positive and negative affect, quality of life, and perceived stress) with PRQ. Emotions (i.e., physical states arising from the body's responses to external stimuli) and feelings (i.e., mental experiences of bodily cues; Scherer and Moors, 2019) are defined as momentary aspects of affective life. On the other hand, core affect is not tied to one specific circumstance, but to a collection of different inputs; hence, it is more stable and shows a certain tendency over time, dependent on numerous intrinsic (physiological and psychological) and extrinsic (interpersonal and sociopolitical) stimuli (Miller, 2011; Stringer, 2013). An individual sees themselves and the world through an “emotional lens” (Kopsov, 2019); hence, quality of life and perceived stress are directly dependent on physiological and cognitive capacity to recognize the impact of external influences and to regulate emotional response (i.e., social-emotional skills). This process represents the bridging function of the affect and well-being as a self-building-block that further dictates the response of the peripheral self-building-block.

A person's adaptive capacity is developed through early interactions with caregivers and relationships with significant others in schoolchildren (Gross, 2013b). Adverse social experiences may hinder the development of social-emotional skills (Lereya et al., 2015; Beutel et al., 2017) or buffer the adverse impact of other environmental planes i.e., of the family (Parra et al., 2018; Yeung and Li, 2019) and community plane (Pashang et al., 2018).

Future research should examine the developmental pathways and relationships between both—trait and state like aspects of affect and well-being, along with their bio-physiological component to be able to understand the bridging role of this self-building-block. This is necessary for comprehensive understanding of individual functioning in SPR and other environmental contexts and could have a vast significance for the youth mental health.

### Behavior and Health as a Peripheral Self-Building-Block

The final category of self-building blocks comprises context specific external behavioral manifestations (clinical and non-clinical) of the self, expressed as a reaction to its interpretation of the environment i.e., peripheral block. In line with previous research (Rueger et al., 2016; Maes et al., 2019), we underline an association between SPR and internalizing spectrum behavior (i.e., depression, somatic symptoms, social anxiety, and withdrawal). However, we find evidence of only a very small association (type C) between SPR and externalizing spectrum behavior (as a generally defined personal construct, distinct from the more specific and relational peer victimization, discussed below). Self-disclosure and prosocial behavior are highly relevant non-clinical behaviors associated with PRQ.

Consistent with the internalizing behaviors as potential determinants of SPR and general interaction principles in our model, such behaviors in youth have been conceptualized as having an interpersonal etiology (Epkins and Heckler, 2011; Madigan et al., 2013). Further, in line with victimization as an important component of the peer environmental plane as discussed below, longitudinal results stipulate a role of victimization in both short-lived and persistent internalizing symptoms (Zwierzynska et al., 2013; Brendgen and Poulin, 2018) linked to suicidal ideation and suicide attempts (Brunstein Klomek et al., 2019; Hinduja and Patchin, 2019). Conversely, SPR have a protective role in the relationship between the negative mental health outcomes of victimization and suicidal ideation (Jenkins et al., 2018). Hence, SPR may act as a protective factor against internalizing symptoms via the already discussed influence on core blocks (e.g., Eisenberg et al., 2005). Indeed, universal school-based interventions, which target social-emotional skills and peer support are found to be promising especially given their potential wide reach (Calear et al., 2016; Bonell et al., 2018).

Future research should commit to exploring the dynamism and interdependence between SPR and peripheral block together with the other self-building-blocks in order to gain more nuanced understanding of clinical and non-clinical behaviors and internalizing and externalizing behaviors. Understanding the mechanisms of environmental influences in these processes and the intersecting roles of the peer and school environments are another important task for researchers.

### Peer Environmental Plane

Peer environment encompasses relationship(s) with at least one peer (i.e., individual related with the person based on any type of similarity with no power or responsibility differential). In our model, experienced inclusion is a key positive correlate of SPR, whilst victimization and peer related stress show the opposite effect. Our results demonstrate only a very small relationship (type C) between deviant behavior in peer group and SPR. However, this construct does not exclusively incorporate deviant behavior toward another peer, which is the case with victimization.

In line with interactions in our model, the negative consequences on long-term well-being of deviant behavior toward peers (i.e., bullying or peer aggression) have been well-documented (e.g., Zwierzynska et al., 2013; Brunstein Klomek et al., 2019). Importantly, such behavior is often rooted in parental prohibition of friendships and parental control (Keijsers et al., 2012) and appears to be resistant to targeted interventions (Barnes et al., 2014; Bonell et al., 2018). This emphasizes the need to consider the links between SPR, conventional relationships (school, teacher, and community) and the corresponding environmental contexts, due to their potential to balance peer influence, and to positively influence peer outcomes (Karcher et al., 2008).

Thus, there is a need for a more nuanced examination of both the deviant behavior (that causes victimization) and its macro (sociopolitical) level, such as cultural norms, values, beliefs, and related polices in order to understand how to buffer the detrimental impact of such behavior within family, school, and peer environmental planes. Furthermore, PR need to be assessed with other conventional relationships in order to capture a comprehensive picture of the social realm and possible causes of externalizing tendencies.

### School Environmental Plane

In line with previous research (Witherspoon et al., 2009) the school surroundings is a key environmental plane for SPR in our model and for well-being in general. As discussed, school belonging and relationship with the teacher (conventional relationships) are protective (Steiner et al., 2019), especially for children faced with adversity in the family (Nichols et al., 2016; Foster et al., 2017).

Consistent with socially mediated reciprocal interactions, school environment provides access to mental health services and opportunities for meaningful parental involvement (e.g., Nichols et al., 2016; Barger et al., 2019). Further, individual perception of the school environment (i.e., positive attitudes toward school and academic engagement) may buffer against deviance in the peer group (Honora and Rolle, 2002) and increase well-being (Pietarinen et al., 2014) via its influence on conventional and unconventional relationships. Finally, the school environment provides one of the most feasible and efficient ways of fostering resilience in children and young people (Ungar et al., 2014; Dray et al., 2017; Bonell et al., 2018). Hence, instead of being on the margins of research agendas, school settings need to be recognized as a potent and cost-effective prevention setting by researchers and policy makers alike.

In terms of advancing research on the school environmental plane, it is important to note that school belonging measures include items on relationships with peers and/or teachers, hampering efforts to measure constituent constructs individually. Hence, future research should make efforts to understand the complementary roles these relationships have within the school environmental plane, advance knowledge on influences of specific sociopolitical factors (e.g., educational and social policies, cultural norms etc.) that shape the dynamics of these relationships, and propose validated and flexible ways of measuring these. Integration of qualitative stakeholder-lead models is crucial for achieving these goals.

### Family Environmental Plane

Family influence weakens during early adolescence. Hence, the relatively peripheral position of the family plane in our model, along with the small percentage of salient results, is not surprising. However, as discussed above—parental attachment, support, and positive parenting are crucial for the development of all self-building blocks (Groh et al., 2012, 2014; Hoeve et al., 2012; Madigan et al., 2013; Orth and Robins, 2019) and may even have a direct influence on SPR. This is in line with principles of interdependence between the self as a whole and environment we propose in our model. Yet, the interactions between the different environmental planes are another important research avenue as well as the understanding or the sociopolitical factors that seem to be especially evident in family environment.

Interventional research has shown that different layers of prevention targeting different environmental planes may influence a child's social and emotional development: systemic interventions focusing on sociopolitical justice and gender equality in schools and communities (e.g., Repetti et al., 2002; Murray et al., 2012) as well as family interventions focusing on interparental relationship, parental mental health and parenting skills (e.g., Coatsworth et al., 2002; Bakermans-Kranenburg et al., 2003; de Graaf et al., 2008). These are important indicators of the significance of the sociopolitical factors for the family environmental plane and the potential interpersonal mechanisms of influences on SPR and well-being that present potent targets for future research.

### Community Environmental Plane

The community environment also yields large relationships with SPR in our model. This encompasses relationships with significant others and the community, which are a meaningful resource for youth (Grossman and Bulle, 2006; Foster et al., 2017). Via these relationships, youths gain access to otherwise unavailable informal resources (e.g., social networks, experiences and knowledge—Grossman and Bulle, 2006) and formal opportunities for meaningful engagement and participation (e.g., in youth organizations) that may increase a sense of personal value, hopefulness, and agency (McLaughlin, 2000).

The notable strength of the association between SPR and quality of relationships with significant others (type A^+^) is consistent with the proposed key role of the core self-building-blocks for youth relationships and well-being, shared developmental pathways and the socially mediated environmental factors on SPR. This is especially important for children from socially disadvantaged families affected by poverty (Sieving et al., 2017) or parental physical and mental illness (Bee et al., 2013), who might be in need for additional adult support and/or role models. Hence, examining interactions between the community and family plane (including interpersonal and sociopolitical factors) and their role in SPR and well-being might be of specific importance for socially disadvantaged early adolescents.

In order to recognize and address complex needs of these young people, researchers need to involve them directly in the research process. This may increase feasibility, youth-friendliness, and ecological validity of work and improve the value and impact of the research results (Checkoway and Richards-Schuster, 2003; Dickson-Hoyle et al., 2018; Hawke et al., 2018). Hence, we underline the necessity of the direct stakeholder involvement for in depth understanding of a role community environmental plane has in SPR and well-being and for the development of acceptable, effective, and sustainable interventions.

### Emerging Virtual Environmental Plane

We found insufficient evidence to include the virtual environment in our model. The only salient finding was a negative association (type A) between PRQ and addictive technology use. However, we were unable to differentiate between usage types (i.e., social media, gaming, and other online contents). The association with the quality of online communication was highly significant but very small (type C).

Indeed, use of social media and television in adolescents may enhance symptoms of depression, whereas the association with gaming is non-significant (Boers et al., 2019). Further, important models of online victimization stipulate a role of internalizing behaviors in increased cyber-victimization (Van Zalk and Van Zalk, 2019); linked to suicidal ideation (Cénat et al., 2019). Yet, these effects can be explained by the upward social comparison hypothesis (Festinger, 1954; i.e., people compare themselves with others who they believe are in a more favorable position, of which more are readily accessible in social media contexts) and the reinforcing spirals hypothesis (Slater, 2007, 2015; i.e., people seek out and select information consistent with their cognitions).

On the other hand, virtual domains (especially instant messaging and social networking sites) can provide an important forum for building social connections in youth (Bourgeois et al., 2014). They can increase the ability to initiate offline friendships (Koutamanis et al., 2013) and social cognition skills (Vossen and Valkenburg, 2016). Hence, the virtual environment has been linked to all youth's relationships (Carroll et al., 2017), yet it remains unacknowledged as a formal dimension of multidimensional social relationship constructs. Further, little is known about the preventive potential of the online setting (Robinson et al., 2016) and complex interventions stretching between the two settings to address on- and offline SPR are completely unaddressed, in spite of the demonstrated overlap between the two relational settings (Reich et al., 2012).

One of the main tasks for future research is to deepen the understanding of the virtual environmental plane as potential determinant of SPR and its interactions with the self-building-blocks and physical environmental planes. This may lead toward development of integrative research informed measures of PR that incorporate virtual environment in a way meaningful for future research on SPR and youth mental health.

### Strengths and Limitations

One of the main strengths of this review is the widely defined search terms and broad inclusion criteria that allowed us to identify a large number of studies and diverse groups of potential determinants. Due to the exclusion of dissertations and failure to identify other unpublished literature, our findings may be prone to a “non-publication bias” (Bassler et al., 2016). However, we addressed this possibility by requesting missing correlations from authors and included secondary and exploratory endpoints that were not crucial for the publication. Furthermore, multiple sensitivity analyses confirmed consistently small to irrelevant publication bias pertinent to our findings. Inclusion of only papers published after 1999 may also introduce bias and result in missing factors or de/increased scores for some variables. However, we decided to focus on a time span with roughly comparable and consistent conditions for youth in order to develop a model pertinent today rather than a historical picture. For example, more than 20 years ago, the communication technologies such as instant messaging, email and social media that transformed social worlds of adolescents (Grinter and Palen, 2002; Nesi et al., 2018a), would not have been a significant aspect of young people's lives and the global sociopolitical context (cultural, economic, political climate) also changed significantly during this period (e.g., Fukuda-Parr, 2004; Ghaemi, 2020).

Another limitation is the presentation of gender-aggregated scores without taking into account gender differences that may introduce significant disparity between early adolescent boys and girls (Rose and Rudolph, 2006). Gender differences might be especially relevant when studying developmental trajectories of the self-building-blocks that need to be examined with respect to relevant sociopolitical context and pertinent gender roles (e.g., Doey et al., 2014; Lavoie et al., 2019; Lewis et al., 2020). Furthermore, we were unable to differentiate between dyadic and peer group relationships, which may influence our results given that the strength of association between certain variables can differ for these two contexts (e.g., Rueger et al., 2016; Narr et al., 2017). Hence, studying dynamics between dyadic and peer group relationships is an important avenue for further research.

The relatively narrow age range in comparison to other similar meta-analyses (Gorrese and Ruggieri, 2013; Gorrese, 2016; Rueger et al., 2016) focusing on early adolescents is a strength of this review. It introduces consistency given the differences in peer relational patterns between early and older adolescent groups (Rueger et al., 2016). We focused on the period between 8 and 14 years because it includes the major stages in the development of SPR during early adolescence, specifically primary school as a key setting in which early PR are established and school transition, which imposes significant challenges for SPR (Waters et al., 2014; Oriol et al., 2017). However, due to the inconsistent definition of early adolescence in the extant literature, some studies with an applicable mean age of participants included wide age ranges, which contaminated our findings with data from outside our target age range. However, a minority (18.3%) of variables included in the meta-analyses had samples in which mean age exceeded 14 years.

We consider the use of self-reported measures of PRQ as a strength of the review given the subjective nature of the PRQ construct (Carroll et al., 2017) and children's demonstrated ability to reliably assess the status of their friendship (Berndt and McCandless, 2013). However, our model was developed primarily from literature relating to typically developing, Caucasian, majority populations in the Western world and therefore has a limited applicability, especially to different cultural contexts and to vulnerable or disadvantaged youth whose perspectives need to be understood in relation to pertinent developmental contexts. Hence, expanding understanding on cultural differences and specific needs of vulnerable young people is imperative for future research. Further, a nuanced examination of peer perspectives and sociometirc measures (van den Berg et al., 2020) in relation self-reports is necessary to gain a complex understanding of early adolescents' social realm.

The employed statistical method also has a few limitations. The Fisher z transformation is mainly derived for Pearson correlation coefficients (Fisher, 1921). Based on the data from the original studies, we were unable to distinguish between Pearson and Spearman coefficients. However, as the minimum sample size for the calculation of the correlation coefficients was 33 (the mean sample size was 504 with a standard deviation of 700), we apply the Fisher z transformation for both Spearman and Pearson correlations in an exploratory way. Further, the statistical heterogeneity (*I*^2^) was consistently high for most variables. This is not surprising as we combined studies with cross-sectional and longitudinal design, different sample sizes, measurement instruments and wide age ranges in some studies. Yet, as the purpose of this meta-analysis is to generate hypotheses in an exploratory way and not to confirm one, the high heterogeneity values are not of concern for the interpretation of the results. Indeed, it is important to allow for heterogeneity through the use of random-effects models, especially when interested in parameter estimation rather than hypothesis testing (Zeng and Lin, 2015).

Given that we included all eligible studies regardless of their methodological quality, which was poor in 95 % of papers, an unavoidable limitation of the present work is the poor quality of the included research. However, the inclusive nature of our review and large number of studies included help to counteract this limitation in the exploratory context of the present review aims. Finally, our findings are based on different numbers of studies—varying from 2 to 71 per variable; hence future research should establish replicability for certain variables.

## Conclusion

The present study is a first step toward an integrated framework for understanding the role of SPR in well-being in early adolescence. By synthetizing the last 20 years of research in the field, we provide evidence on the key role of SPR in early adolescence and its interrelatedness with all aspects (internal and external) of early adolescent's lives. We propose an evidence-based model of SPR that may serve as a framework for future harmonization of theoretical and interventional research efforts toward evidence based interventions. The model describes the central importance of identity in PR, which interacts with individual factors including social-emotional skills, core affect, general perceptions of well-being and specific behaviors to form an organized self. All such self-building-blocks are further influenced by environmental planes, including peer, school, family, and community environments to build an integrated system that functions as an organized whole. In the context of the exhaustive approach to collating evidence examining factors affecting PR, we discuss the general (related to PR aspects, gender, culture etc.) and specific (related to the identified model components) future research avenues that are necessary for refinement and validation of the proposed model for the purpose of overcoming the current fragmentation in the field. To achieve this ambitious goal we advocate use of adequate and validated measurement instruments, mixed—methods techniques, an integrative approach to designing quantitative studies, process models that allow understanding of complex mechanisms of interdependence, and when possible longitudinal designs. The scope of such an endeavor demands interdisciplinary expertise, direct stakeholder involvement in the research process and the combination of top-down (empirical) and bottom –up (interventional) data. Ultimately, we hope that our approach will allow advancement toward evidence-based integrated ecological prevention programs that stretch across multiple environmental planes in a structured and coordinated manner to provide optimal sociopolitical climate that supports relevant interpersonal relationships (especially SPR) necessary for the holistic development of the resilient self.

## Data Availability Statement

The datasets presented in this study can be found in online repositories. The names of the repository/repositories and accession number(s) can be found in the article/Supplementary Material.

## Author Contributions

MM was a leading author on the project involved in funding acquisition, conceptualization, methodology design, data collection (conducted searches), extraction, analysis, validation, visualization, and writing of an original draft. KW and BS were joint senior authors involved in funding acquisition, conceptualization, methodology design, supervision of the data analyses, and writing - commenting on the original draft. IK and KS were involved in methodology design, data extraction, analysis, and validation. SZ performed statistical analysis and supported writing of the original draft. MA provided external supervision on the project. All authors contributed to the article and approved the submitted version.

## Conflict of Interest

The authors declare that the research was conducted in the absence of any commercial or financial relationships that could be construed as a potential conflict of interest.

## Supplementary Material

The online supplement is available at the Figshare repository [10.6084/m9.figshare.7988417] and it includes:

Supplementary Table 1 Characteristics of included studies.

Supplementary Table 2 Measures of peer relationship quality.

Supplementary Table 3 List of excluded studies with reasons.

Supplementary Figure 1 Conceptual triangulation.

Supplementary Figure 2 Histogram of estimated correlations coefficients.

Supplementary Figures 3–95 Forest plots for all variables.

Supplementary Figures 96–219 Publication bias funnel plots.

Supplementary Figure 220 Geographic data.

Supplementary Document 1 Search strategy.

Supplementary Document 2 Risk of bias (RoB) tools adaptation.

Supplementary Document 3 List of included studies.

Supplementary Document 4 PRISMA checklist (2009).

Supplementary Document 5 PRISMA flow diagram (2009).

Supplementary Data set Meta-analysis data set.
